# Effect of disease modifying anti-rheumatic drugs on major cardiovascular events: a meta-analysis of randomized controlled trials

**DOI:** 10.1038/s41598-021-86128-y

**Published:** 2021-03-23

**Authors:** Shivshankar Thanigaimani, James Phie, Smriti Murali Krishna, Joseph Moxon, Jonathan Golledge

**Affiliations:** 1 The Queensland Research Centre for Peripheral Vascular Disease (QRC-PVD), College of Medicine and Dentistry, James Cook University, Townsville, QLD 4811 Australia; 2 The Australian Institute of Tropical Health and Medicine, James Cook University, Townsville, QLD Australia; 3 The Department of Vascular and Endovascular Surgery, Townsville University Hospital, Townsville, QLD Australia

**Keywords:** Antibody therapy, Rheumatoid arthritis

## Abstract

Disease modifying anti-rheumatic drugs (DMARDs) were developed to treat joint inflammation. There is growing evidence that anti-inflammatory drugs prevent major cardiovascular events (MACE). The aim of this systematic review and meta-analysis was to examine whether DMARDs reduce the risk of MACE. A systematic literature search was performed to identify randomized controlled trials (RCTs) testing the effect of DMARDs on cardiovascular events. The primary outcome was MACE defined as the first occurrence of non-fatal myocardial infarction (MI), non-fatal stroke or cardiovascular death. Secondary outcomes were myocardial infarction or stroke alone and all-cause mortality. Safety was assessed by fatal or life threatening infection. Meta-analyses were performed using random effect models and reported as risk ratios (RR) and 95% confidence intervals (CI). Study quality and publication bias were assessed using the Cochrane Collaboration’s tool for assessing risk of bias and funnel plots. Twelve RCTs involving 18,056 participants testing three different DMARDs subclasses (Tumor Necrosis Factor inhibitors—4 trials; Janus Kinase inhibitors—5 trials; Interleukin inhibitors—3 trials) were included. Meta-analysis suggested that none of the DMARD subclasses had any effect on MACE, MI alone, stroke alone, risk of fatal or life threatening infection or death. Risk of bias was high, low and unclear in five, six and one studies respectively. Funnel plots suggested a low possibility of publication bias. This meta-analysis suggests that DMARDs do not affect the incidence of MACE. More trials are needed for firm conclusions.

## Introduction

Inflammation is strongly implicated in atherosclerosis progression and associated thrombosis^1,2^. High circulating concentrations of inflammatory biomarkers, such as C-reactive protein, are associated with a high incidence of cardiovascular (CV) events and have been used to identify people for preventative therapy, such as statin prescription^3–5^. The Canakinumab Anti-inflammatory Thrombosis Outcome Study (CANTOS) showed that people randomly allocated to a monoclonal antibody blocking interleukin-1β (canakinumab) had a reduced incidence of major cardiovascular events (MACE, i.e. myocardial infarction (MI), stroke or cardiovascular death) compared to those allocated to placebo^6^. This finding suggests that other anti-inflammatory drugs may also have a therapeutic role in prevention of cardiovascular events.

Disease modifying anti-rheumatic drugs (DMARDs) were developed primarily to treat joint inflammation^7–9^. Due to their powerful anti-inflammatory effects, it is plausible that DMARDs may also prevent cardiovascular events. Inhibition of cytokines, such as tumor necrosis factor (TNF) and interleukins (ILs), and janus kinase (JAK) have been reported to limit inflammatory mechanisms implicated in athero-thrombosis through studies in animal models^10–12^. Most commonly prescribed biologic and targeted synthetic DMARDs such as TNF-alpha (α) inhibitors, IL-1 beta (β) & IL-12/23 inhibitors and JAK-1/2 inhibitors have been tested in randomized controlled trials within a variety of different populations that are at risk of MACE, such as people with rheumatoid arthritis and psoriasis^13–17^. Prior clinical trials report both positive^6^ and negative findings^18,19^ on the effect of DMARDs on MACE. Further, there is no consensus on whether all DMARDs are effective in reducing MACE, with prior systematic reviews reporting both positive^20,21^ and negative^22,23^ findings by comparing between biologic and conventional synthetic DMARDs and also within subclasses of biologic DMARDs.

Prior meta-analyses have been restricted to specific disease populations, such as people with psoriasis^24^ or rheumatoid arthritis^25^, limiting the power to test the effect of DMARDs on MACE. Previous systematic reviewers have also incorporated observational data together with findings from randomized controlled trials limiting the ability to interpret findings due to lack of comparison to placebo controlled groups^22,25–31^. Furthermore, randomized trials specifically examining the effect of DMARDs on MACE have been published since these previous meta-analyses were reported; meaning that more up to date data are available but have not been systematically appraised^6,14,32,33^. In view of this, and past lack of consensus and limitations of previous systematic reviews, there is a need for an up to date comprehensive meta-analyses. This systematic review and meta-analysis aimed to provide an up to date test of whether DMARDs reduce the risk of cardiovascular events, using data from all available randomized controlled trials.

## Methods

### Search strategy and eligibility criteria

The systematic review was performed according to the Preferred Reporting the Items for Systematic Review and Meta-Analysis (PRISMA) statement and registered in the PROSPERO database (Registration Number: CRD42020166140). The literature search was conducted by one author (ST). The databases PubMed, Cochrane Central Register for Controlled Trials and Scopus were searched on 12th January 2021. The search strategy included the synonyms or similar terms of "Cardiovascular events" AND "DMARDs" AND “Randomized controlled trials” (See supplementary material for full search terms). No date and language restriction were applied. This review was restricted to biologic and targeted synthetic DMARDs that target TNF-α, IL-1β, IL-12/23 and JAK1/2. To be eligible for inclusion, studies had to adopt: A randomized controlled design; test drugs in the DMARD class that target TNF, IL or JAK; include a placebo control group; and either reported the number of CV events (at least one of MACE, MI alone or stroke) or all-cause mortality. Both full text and abstracts of studies meeting entry criteria were included as long as minimum data were available either within the report, within the clinical trial registration entry or by contacting the corresponding author. Minimum data included DMARD tested, dose used, mean age of patients in control and intervention groups, duration of treatment and reported number of MACE, MI, stroke or death in both intervention and control groups. Exclusion criteria included trials adopting an open label or non-randomized design and studies where minimum data were not available. Included articles were identified by one author (ST) and reviewed by two more authors (JP, SMK) to confirm that they met entry criteria. Any discrepancies were resolved by discussion with another author (JM).

### Data extraction and outcomes

Data were extracted on a customized spreadsheet by four authors (ST, JP, SMK, JG). Any inconsistencies were resolved through discussion with another independent author (JM). The primary outcome of this study was MACE incidence, defined as the first occurrence of any non-fatal MI or non-fatal stroke or cardiovascular death. Death occurring due to MI, stroke or any other cardiovascular related conditions was defined as cardiovascular death. Secondary outcomes were the incidence of MI, stroke or cardiovascular death alone, or all-cause mortality. Safety was assessed through the incidence of fatal or life threatening infection or infections requiring hospital admission. Outcome data were reported relative to the number of patients in whom results were reported for. The following information was also extracted from the included trials: Primary diagnosis of the population, cardiovascular risk factors, sample size for both control and intervention groups, name, dose and type of DMARD drug tested, entry and exclusion criteria for the trial, duration of treatment period, age, sex, history of MI and stroke and use of DMARDs other than the drug being tested (including TNF-α or receptor inhibitors, IL-1β or IL-12/23 inhibitors and JAK-1/2 inhibitors) at baseline.

### Quality assessment

Three authors independently assessed the risk of bias of all included studies using the Cochrane Collaboration’s tool which assessed key aspects of the reports including guideline definitions for included patient groups, random sequence generation, blinding of participants and assessors, statistical sample size estimate, reporting the number of patients who completed the study, percentage of patients who had incomplete data, a clear primary outcome, intent-to-treat analysis and other biases^34^. Any inconsistencies were resolved through discussion between the authors until a consensus was reached. Each aspect was rated as low, high or unclear risk of bias. Each trial was then given an overall rating of risk of bias. If any aspect of the trial was considered high (or unclear) risk of bias this overall rating was given. A low risk of bias rating required all aspects to be given this assessment (Supplementary Table 14).

### Data analysis

Meta-analyses were performed for any of the primary or secondary outcomes where data were available from at least three trials. The main analyses pooled data from individual DMARD classes, specifically, TNF-α inhibitors, JAK-1/2 inhibitors or IL-1β, IL-12/23 inhibitors, where data for one or more outcomes were available from a minimum of three trials. In trials that had no events in either one of the groups, ‘zero’ event was imputed by adding one event equally to all groups in the study. Due to the small number of eligible studies, meta-analyses were performed using the inverse variance method with random effects models and applied Sidik-Jonkman method with Hartung‐Knapp modification to estimate the between-study variance (tau2)^35^. This estimator uses the Q-profile method to provide a conservative and broader CI to minimize the risk of false positive results. Random effects analyses were preselected with an anticipation of high heterogeneity between studies. Statistical heterogeneity between studies was assessed using the I^2^ statistic and interpreted as low (0 to 49%), moderate (50 to 74%) or high (75 to 100%)^36^. The contribution of each study to the pooled estimates of outcome measures were assessed by performing leave-one-out sensitivity analyses where individual studies were excluded to inspect if they influenced the overall estimated effects. Publication bias was assessed by funnel plots comparing the overall effect size of each study to the standard error of its log-transformed effect^37^. Meta-analysis was conducted using ‘meta’ package and sensitivity analysis was performed using ‘dmetar’ package of R software version 3.4.4. All statistical tests were two-sided and p value of ≤ 0.05 was considered significant.

## Results

### Included trials and participants

**Figure 1:**
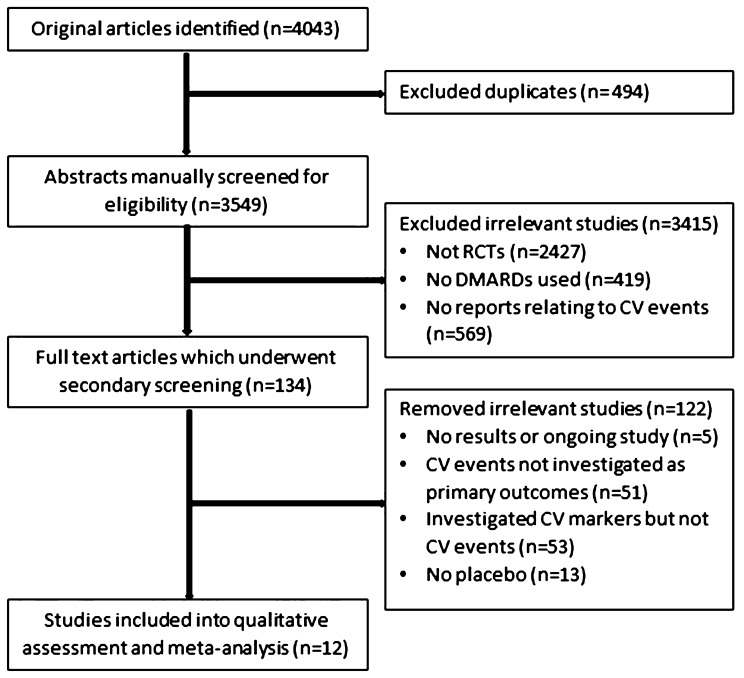
Preferred Reporting Items of Systematic Review and Meta-analyses (PRISMA) flow diagram. A total of 4043 studies were screened and 12 Randomized trials were included for analyses.

The literature search identified 4043 unique records of which 12 trials including a total of 18,056 participants ultimately met the inclusion criteria (Fig. 1). Four trials tested TNF-α inhibitors (Infliximab or Certolizumab pegol) or a TNF receptor inhibitor (Etanercept) in 3612 participants^18,38–40^. Five trials tested JAK-1/2 inhibitors (Upadacitinib or Baricitinib or filgotinib) in 2819 people^14,33,41–43^. Three trials tested IL inhibitors (Briakinumab or Canakinumab or Anakinra) in 11,625 participants^6,15,44^. Three trials were conducted in the USA^38,39,44^ and the other nine trials recruited people from multiple countries^6,14,15,18,33,40–43^. The primary diagnoses and risk factors of the participants involved in the included trials were heterogeneous (Supplementary Table 1 and 2). People with rheumatoid arthritis were included in trials testing Etanercept^39^, Upadacitinib^14,33^, Baricitinib^41,42^, Certolizumab pegol^40^ and Filgotinib^43^. People with heart failure were included in trials testing Infliximab^38^ and Etanercept^18^. People with psoriasis were included in trials testing Briakinumab^15^ and MI patients were included in trials testing Canakinumab^6^ and Anakinra^44^. The medical management of the included participants, such as prescription of statins, anti-platelet and anti-hypertensive medications, was poorly and variably reported (Table 1). The follow-up periods in the trials ranged between 12 and 54 weeks (Table 1). Eligibility criteria, follow-up and study centers are shown in Supplementary Table 2. The outcomes of included studies are shown in Table 2.

**Table 1:** Baseline patient characteristics.

Author, Year	Group/dose (no. of patients)	Duration of follow-up (weeks)	Age (Years)	Male gender (%)	Previous history of CHF (%)	Use of Biologic DMARDs at baseline	Statins (%)	Antithrombotic agent or anticoagulants (%)	Anti-hypertensive drugs (%)
**Tumor necrosis factor inhibitors**									
Chung et al., 2003	Placebo (n = 49)	28	60 ± 12.0	76.0	100.0	No	NR	NR	84.0*
	Inflximab 5 mg/kg (n = 50)		62 ± 15.0	86.0	100.0	No	NR	NR	80.0*
	Infliximab 10 mg/kg (n = 51)		62 ± 13.0	84.0	100.0	No	NR	NR	80.0*
Emery et al., 2015^b^	Placebo (n = 219)	52	51.3 ± 13.2	20.1	NR	NR	NR	NR	NR
	Certolizumab pegol (n = 660)		50.5 ± 13.6	24.4	NR	NR	NR	NR	NR
Mann et al., 2004^a^	Placebo (n = 682)	24	64.6 ± 10.8	76.0	100.0	NR	NR	NR	83.5*
	Etanercept QW (n = 375)		64.8 ± 10.3	77.0	100.0	NR	NR	NR	83.0*
	Etanercept BIW (n = 683)		62.95 ± 11.2	79.0	100.0	NR	NR	NR	82.0*
	Etanercept TIW (n = 308)		62.4 ± 11.0	81.0	100.0	NR	NR	NR	79.0*
Weisman et al., 2007^d^	Placebo (n = 269)	16	59.3 (23–85)	21.9	32.0	No	NR	NR	NR
	Etanercept (n = 266)		60.6 (19–84)	27.8	32.7	No	NR	NR	NR
**Janus Kinase inhibitors**									
Burmester et al., 2018	Placebo (n = 221)	12	56 ± 12.2	25.0	NR	No	NR	NR	NR
	Upadacitinib 15 mg(n = 221)		55.3 ± 11.5	18.0	NR	No	NR	NR	NR
	Upadacitinib 30 mg(n = 219)		55.8 ± 11.3	21.0	NR	No	NR	NR	NR
Genovese et al., 2018	Placebo (n = 169)		57.6 ± 11.4	15.0	NR	NR	NR	NR	NR
	Upadacitinib 15 mg(n = 164)	12	56.3 ± 11.3	16.0	NR	NR	NR	NR	NR
	Upadacitinib 30 mg(n = 165)		57.3 ± 11.6	16.0	NR	NR	NR	NR	NR
Genovese et al., 2016	Placebo (n = 176)	24	56 ± 11.0	18.0	NR	Yes	NR	NR	NR
	Baricitinib 2 mg (n = 174)		55 ± 11.0	21.0	NR	Yes	NR	NR	NR
	Baricitinib 4 mg (n = 177)		56 ± 11.0	16.0	NR	Yes	NR	NR	NR
Dougados et al., 2017	Placebo (n = 228)	12	51 ± 13.0	17.0	NR	Yes	NR	NR	NR
	Baricitinib 2 mg (n = 229)		52 ± 12.0	20.0	NR	Yes	NR	NR	NR
	Baricitinib 4 mg (n = 227)		52 ± 12.0	18.0	NR	Yes	NR	NR	NR
Genovese et al., 2019	Placebo (n = 148)	24	56 ± 12.1	18.2	NR	Yes	NR	NR	NR
	Filgotinib 100 mg (n = 153)		56 ± 12.0	22.2	NR	Yes	NR	NR	NR
	Filgotinib 200 mg (n = 148)		56 ± 12.5	18.4	NR	Yes	NR	NR	NR
**Interleukin inhibitors**									
Gordon et al., 2012	Placebo (n = 484)	12	45.1 ± 13.5	70.9	0.2	No	NR	NR	NR
	Briakinumab 200 mg (n = 981)		45.7 ± 13.2	67.9	0.4	No	NR	NR	NR
Ridker et al., 2017	Placebo (N = 3344)	48	61.1 ± 10.0	74.1	21.6	NR	91.1	95.3	79.8**
	Canakinumab 50 mg (n = 2170)		61.1 ± 10.1	75.1	20.8	NR	91.7	94.9	79.3**
	Canakinumab 150 mg (n = 2284)		61.2 ± 10.0	74.8	20.9	NR	90.6	94.6	79.8**
	Canakinumab 300 mg (n = 2263)		61.1 ± 10.1	74.4	23.1	NR	91.1	95.1	79.6**
Abbate et al., 2020^d^	Placebo (n = 35)	54	56 (51–65)	85.7	NR	NR	NR	20.0^	NR
	Anakinra Once daily (n = 33)		53 (49–62)	72.7	NR	NR	NR	15.0^	NR
	Anakinra twice daily (n = 31)		55 (45–61)	83.9	NR	NR	NR	13.0^	NR

NR, Not reported; CHF, Congestive Heart Failure.

*, Angiotensin converting enzyme inhibitors; **, Renin angiotensin system inhibitors; ^, Clopidogrel.

^a^Study was designed as two independent clinical trials. Placebo and BIW data were pooled together for analysis.

^b^Study characteristics data taken from another published article mentioned in clinical trials database.

^d^Age is reported as median (min–max).

**Table 2:** Outcome data from all included studies testing the effect of DMARDs.

Reference	Groups	Lost to follow up	MACE	MI	Stroke	Serious infections	All-cause mortality
**TNF inhibitors**							
Chung et al. 2003^a^	Placebo (n = 49)	0	NR	NR	NR	1	4
	Infliximab 5 mg/kg (n = 50)	0	NR	NR	NR	3	4
	Infliximab 10 mg/kg (n = 51)	0	NR	NR	NR	4	8
Emery et al., 2015^c^	Placebo (n = 219)	2	2	0	2	7	1
	Certolizumab pegol (n = 660)	1	5	2	2	20	2
Mann et al., 2004^a,^*	Placebo (n = 682)	0	NR	NR	NR	13	77
	Etanercept QW (n = 375)	0	NR	NR	NR	4	22
	Etanercept BIW (n = 683)	0	NR	NR	NR	14	82
	Etanercept TIW (n = 308)	0	NR	NR	NR	7	61
Weisman et al., 2007^b^	Placebo (n = 269)	0	7	NR	NR	10	NR
	Etanercept (n = 266)	0	13	NR	NR	8	NR
**JAK inhibitors**							
Burmester et al., 2018	Placebo (n = 221)	0	0	0	0	1	0
	Upadacitinib 15 mg(n = 221)	0	0	0	0	1	0
	Upadacitinib 30 mg(n = 219)	0	1	0	1	3	0
Genovese et al., 2018^d^	Placebo (n = 169)	0	0	0	0	0	0
	Upadacitinib 15 mg(n = 164)	0	1	0	1	1	0
	Upadacitinib 30 mg(n = 165)	0	1	0	0	4	1
Genovese et al., 2016	Placebo (n = 176)	0	0	0	0	5	0
	Baricitinib 2 mg (n = 174)	0	0	0	0	4	0
	Baricitinib 4 mg (n = 177)	0	2	1	1	6	1
Dougados et al., 2017	Placebo (n = 228)	0	2	1	1	4	2
	Baricitinib 2 mg (n = 229)	0	0	0	0	2	0
	Baricitinib 4 mg (n = 227)	0	0	0	0	4	0
Genovese et al., 2019	Placebo (n = 148)	1	1	0	1	2	0
	Filgotinib 100 mg (n = 153)	1	1	1	0	3	0
	Filgotinib 200 mg (n = 148)	1	0	0	0	1	0
**IL inhibitors**							
Gordon et al., 2012^d^	Placebo (n = 484)	0	0	0	0	5	0
	Briakinumab (n = 981)	0	5	3	1	1	1
Ridker et l, 2017	Placebo (N = 3344)	0	535	292	92	342	375
	Canakinumab 50 mg (n = 2170)	0	313	169	58	230	228
	Canakinumab 150 mg (n = 2284)	0	320	159	63	258	238
	Canakinumab 300 mg (n = 2263)	4	322	174	51	265	239
Abbate et al., 2020^c^	Placebo (n = 35)	0	3	1	1	5	1
	Anakinra OD (n = 33)	0	2	1	1	3	0
	Anakinra TD (n = 31)	0	1	1	0	6	0

^a^CV death used for MACE calculation is death or hospitalization due to CHF.

^b^MACE calculated from percentage and included heart failure, coronary artery disease, MI and cerebrovascular events. No individual event data was given.

^c^Data taken from clinical trial page.

^d^Placebo group is being randomized into intervention after 12 weeks. Therefore, we considered only data until 12 weeks.

MACE, Major adverse cardiovascular events; MI, Myocardial infarction; CV death, Cardiovascular death; NR, Not reported.

## MACE

Ten studies reported the effect of a DMARD on the incidence of MACE^6,14,15,33,39–44^. Two trials reported that Briakinumab and Baricitinib increased the incidence of MACE^15,41^. Seven trials reported that Upadacitinib, Anakinra, Baricitinib, Filgotinib and Certolizumab pegol had no effect on MACE^14,33,39,40,42–44^ and one trial reported a significant reduction in MACE with Canakinumab^6^.

**Figure 2:**
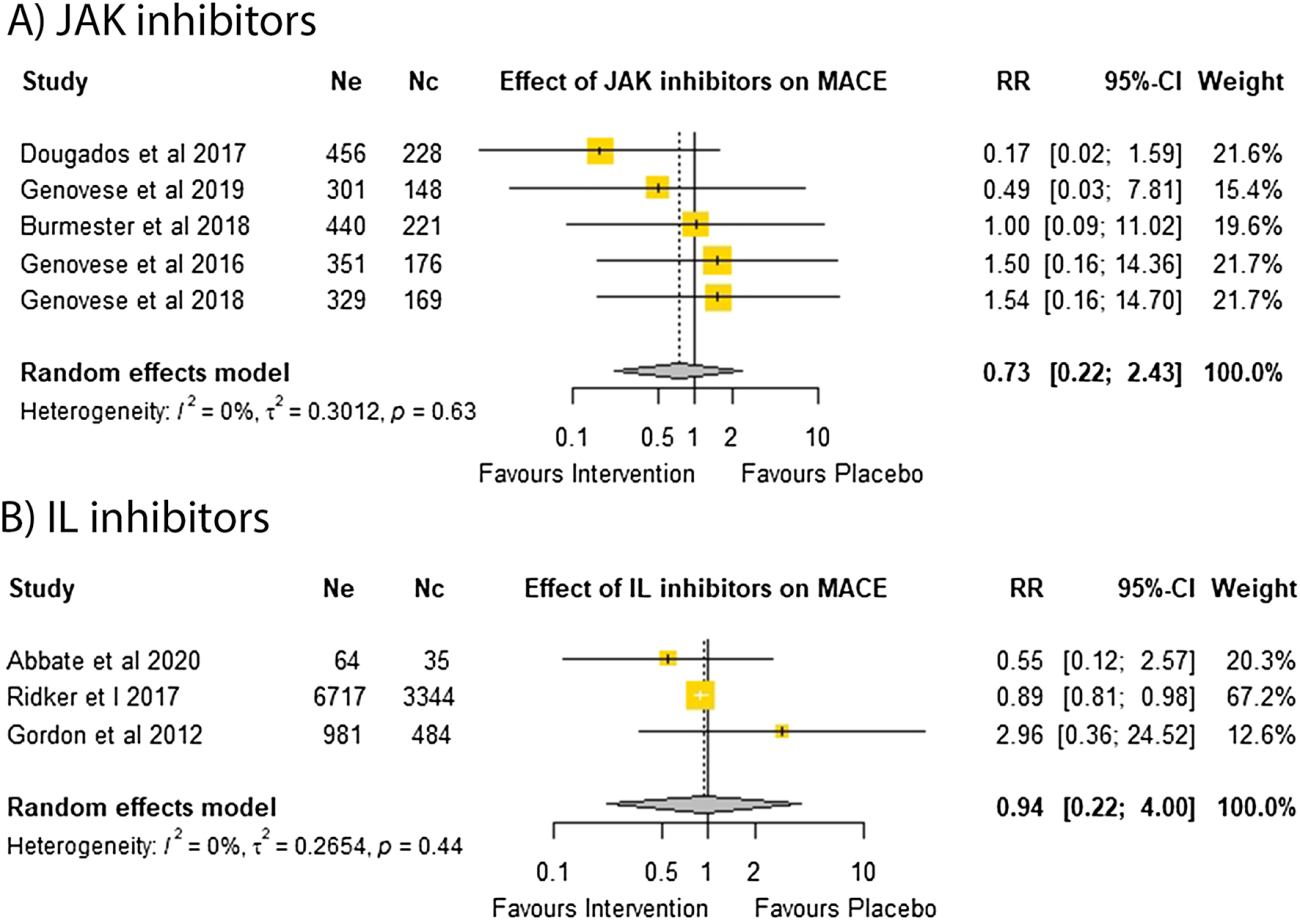
Effect of DMARDs on major cardiovascular events.

A meta-analysis of five trials testing JAK inhibitors in 2819 participants (RR = 0.73; 95% CI 0.22, 2.43)^14,33,41–43^ and three trials testing IL inhibitors in 11,625 participants (RR = 0.94; 95% CI 0.22, 4.00)^6,15,44^ suggested these drugs did not significantly affect the relative risk of MACE with low heterogeneity between the included studies (Fig. 2). Leave one out sensitivity analyses found that the outcomes were consistent (Supplementary Tables 3 and 4). Funnel plots suggested no publication bias (Supplementary Fig. 1). Insufficient trials testing TNF inhibitors reported MACE to qualify for meta-analysis.

### MI alone

Six included trials reported the incidence of MI alone. Three trials testing Certolizumab pegol, Baricitinib and Anakinra reported no significant effect on the incidence of MI^40,42,44^. Two trials reported that briakinumab and baricitinib increased the incidence of MI^15,41^. One trial reported that canakinumab significantly reduced the incidence of MI^6^.

**Figure 3:**
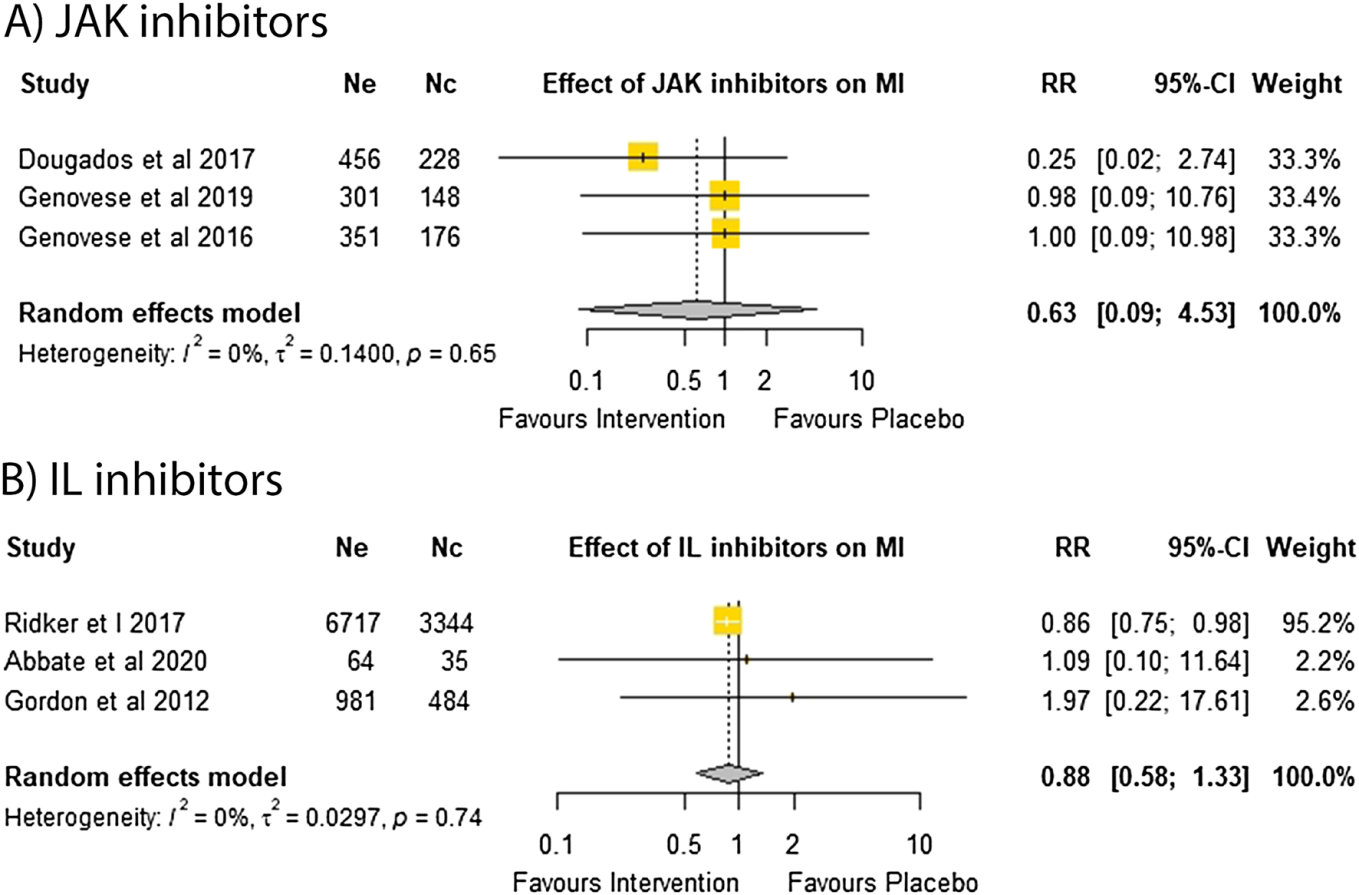
Effect of DMARDs on myocardial infarction alone.

A meta-analysis of three trials testing the effect of JAK inhibitors in 1660 participants suggested no significant effect on the risk of MI (RR = 0.63; 95% CI 0.09, 4.53)^14,41,42^. A meta-analysis of three trials testing the effect of IL inhibitors in 11,625 participants suggested no significant effect on the risk of MI (RR = 0.88; 95% CI 0.58, 1.33)^6,15,44^ (Fig. 3). Sensitivity analyses suggested that the results were robust (Supplementary Tables 5, 6). Funnel plots suggested no publication bias in trials testing JAK inhibitors, but was noted to be asymmetrical in the meta-analysis on IL inhibitors (Supplementary Fig. 2). Insufficient trials testing TNF inhibitors reported MI alone to qualify for meta-analysis.

### Stroke alone

**Figure 4:**
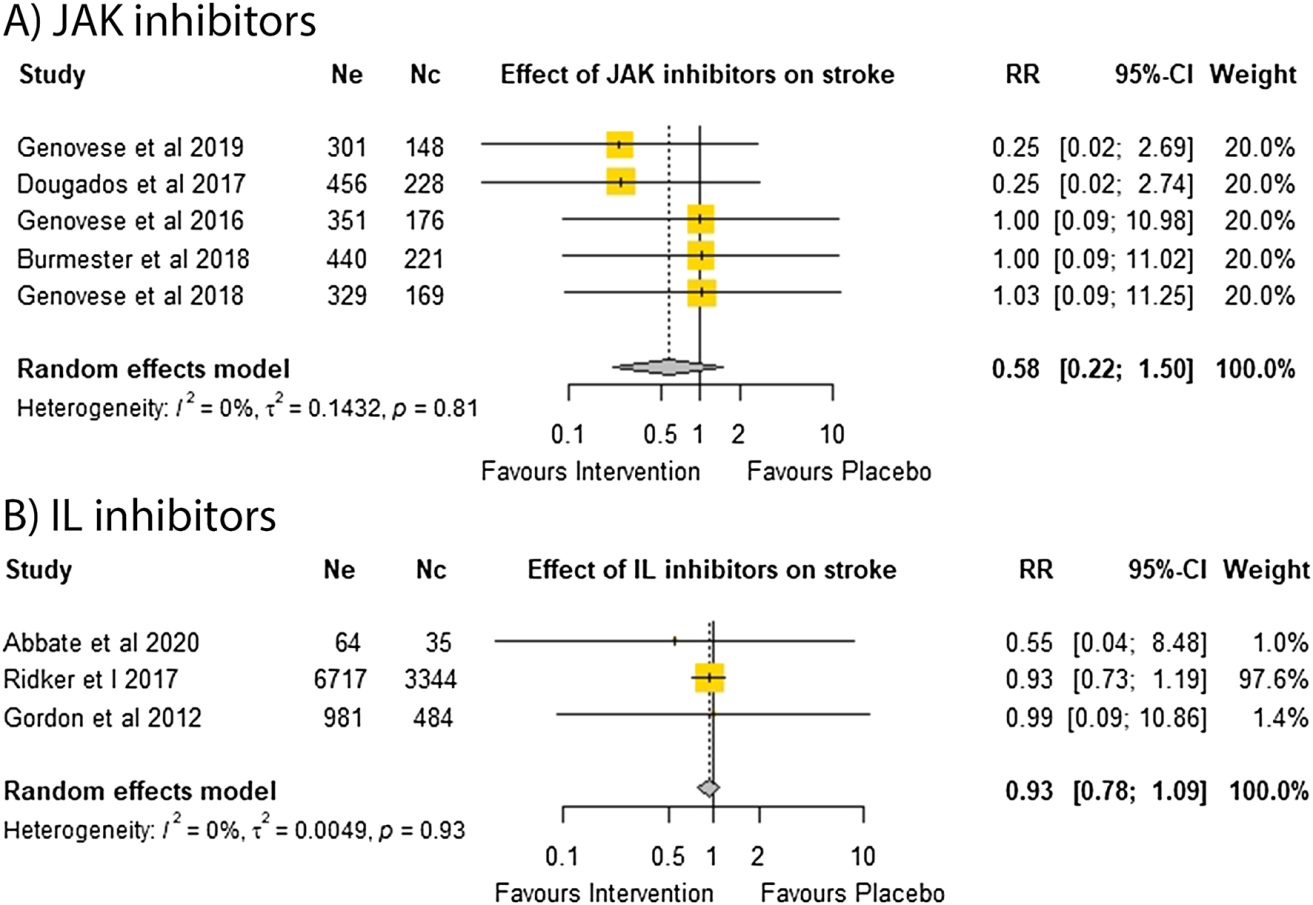
Effect of DMARDs on stroke alone.

Eight of the trials testing DMARDs and all reported no significant effect on the incidence of stroke alone^6,14,15,33,40–42,44^. Meta-analysis of five trials testing JAK inhibitors in 2819 participants (RR = 0.58; 95% CI 0.22, 1.50)^14,33,41–43^ and three trials testing IL inhibitors in 11,625 participants (RR = 0.93; 95% CI 0.78, 1.09)^6,15,44^ suggested that DMARDs had no significant effect on the relative risk of stroke (Fig. 4). Sensitivity analyses and funnel plots showed that the findings were consistent and removal of individual trials did not change the overall results (Supplementary Fig. 3 and Supplementary Tables 7 and 8). Insufficient trials testing TNF inhibitors reported the outcome of stroke alone to qualify for a meta-analysis.

### Cardiovascular death alone

A total of three trials reported cardiovascular death alone^6,15,39^. One trial tested TNF inhibitors in 535 participants and reported 1 and 3 cardiovascular deaths in participants’ allocated placebo and etanercept respectively^39^. Two trials tested IL inhibitors in 11,526 participants and reported 182, 319 and 1 cardiovascular deaths in participants’ allocated placebo, canakinumab and briakinumab respectively^6,15^. Insufficient trials reported this outcome to qualify for meta-analyses.

### Death by any cause

**Figure 5:**
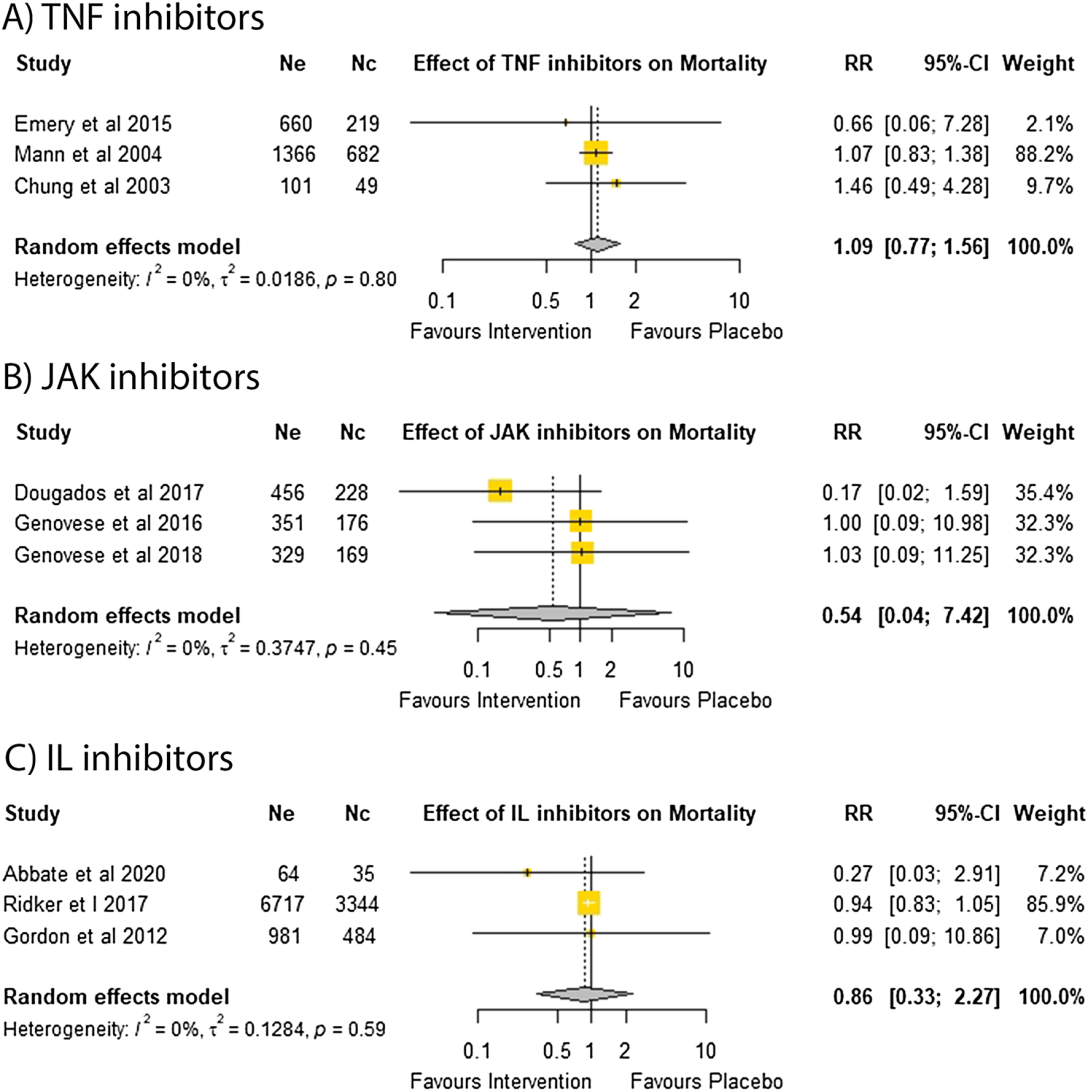
Effect of DMARDs on all-cause mortality.

All 12 trials reported that the DMARDs tested did not significantly affect all-cause mortality^6,14,15,18,33,38–44^. Three trials reported no deaths in both treatment and placebo groups, therefore, the remaining nine trials were eligible for meta-analysis^33,39,43^. Meta-analyses of three trials testing TNF inhibitors in 3077 participants (RR = 1.09, 95% CI 0.77, 1.56)^18,38,40^, JAK inhibitors in 1709 participants (RR = 0.54; 95% CI 0.04, 7.42)^14,41,42^ and IL inhibitors in 11,625 participants (RR = 0.86; 95% CI 0.33, 2.27) suggested that DMARDs did not significantly affect the relative risk of death (Fig. 5). In addition, sensitivity analyses and funnel plots suggested that the results were consistent and unlikely to be subject to publication bias (Supplementary Tables 9–11 and Supplementary Fig. 4).

### Serious or severe infection

All 12 trials reported a non-significant higher incidence of serious or severe infections within people allocated DMARDs^6,14,15,18,33,38–44^. The meta-analyses of four trials testing TNF inhibitors in 3612 participants (RR = 1.00; 95% CI 0.53, 1.90)^6,18,38,40^, five trials testing JAK inhibitors in 2819 participants (RR = 1.12; 95% CI 0.61, 2.07)^14,33,41–43^ and three trials testing IL inhibitors in 11,625 participants (RR = 0.64; 95% CI 0.03, 11.91)^6,15,44^ suggested that DMARDs did not significantly increase the relative risk of serious or severe infections (Supplementary Fig. 5). Sensitivity analyses suggested that the results were consistent and removal of individual studies did not affect the findings (Supplementary Tables 12–14). Funnel plots suggested no publication bias (Supplementary Fig. 6).

### Risk of bias

Overall, six studies had a low risk of bias^6,14,38,41,42,44^, one study had an unclear risk of bias^15^ and the remaining five studies had a high risk of bias^18,33,39,40,43^. Limitations noted included the absence of intent-to-treat analyses^18,33,39,40,43^ and not reporting the primary diagnosis of patients^15^ (Supplementary Table 15 and Table 3). Funnel plots suggested no publication bias (Supplementary Figs. 1, 2, 3, 4 and 6).

**Table 3:** Quality assessment summary of all included studies.

Author	Patient subset definition	Mentioned the number of patients who completed the study	Random sequence generation	Blinding of participants/ personnel	Blinding of assessors	Sample size estimate	Incomplete outcome data (> 10% loss)	Clear primary outcome	ITT	Other biases	Total risk of bias
**Tumor necrosis factor inhibitors**											
Chung et al. 2003	(+)	(+)	(+)	(+)	(+)	(+)	(+)	(+)	(+)	(+)	Low
Emery et al. 2015	(+)	(+)	(+)	(+)	(+)	(+)	(+)	(+)	(−)	(+)	High
Mann et al. 2004	(+)	(+)	(+)	(+)	(+)	(+)	(+)	(+)	(−)	(+)	High
Weisman et al. 2007	(+)	(+)	(+)	(+)	(+)	(+)	(+)	(+)	(−)	(+)	High
**Janus kinase inhibitors**											
Burmester et al. 2018	(+)	(+)	(+)	(+)	(+)	(+)	(+)	(+)	(−)	(+)	High
Genovese et al. 2018	(+)	(+)	(+)	(+)	(+)	(+)	(+)	(+)	(+)	(+)	Low
Genovese et al. 2016	(+)	(+)	(+)	(+)	(+)	(+)	(+)	(+)	(+)	(+)	Low
Dougados et al. 2017	(+)	(+)	(+)	(+)	(+)	(+)	(+)	(+)	(+)	(+)	Low
Genovese et al. 2019	(+)	(+)	(+)	(+)	(+)	(+)	(+)	(+)	(−)	(+)	High
**Interleukin inhibitors**											
Gordon et al. 2012	(?)	(+)	(+)	(+)	(+)	(+)	(+)	(+)	(+)	(?)	Unclear
Ridker et l 2017	(+)	(+)	(+)	(+)	(+)	(+)	(+)	(+)	(+)	(+)	Low
Abbate et al. 2020	(+)	(+)	(+)	(+)	(+)	(+)	(+)	(+)	(+)	(?)	Low

### Discussion

The main finding of this meta-analysis was that the three DMARD drug classes tested had no significant effect on the incidence of MACE, MI alone, stroke alone and mortality and the findings were robust in sensitivity analyses. One of the included trials, testing the IL inhibitor Canakinumab, reported a significant reduction in MACE but this finding could not be replicated in the meta-analysis which included all IL inhibitor trials identified^6^. It should be acknowledged however that the analyses are limited by the number of available trials and heterogeneity of the included populations and further trials are needed for robust conclusions. It was not possible to perform meta-analyses for the outcome of cardiovascular related mortality alone as this was infrequently reported.

The recent European League Against Rheumatism (EULAR) task force recommendations suggested that there was no increase in MACE or cancer but an increased risk of infection with the use of biological (b)DMARDs as compared with conventional synthetic (cs)DMARDs^45^. The meta-analysis suggested that IL inhibitors did not have any effect on the incidence of MACE or the risk of serious or severe infections similar to the current study. These findings suggest that DMARDs may be safe to use, although longer trials are needed. Many of the included trials were very short meaning conclusions on safety should be guarded.

Most of the included trials did not examine cost effectiveness. Data from the Finnish registry suggested that bDMARDs were not cost-effective due to a high incremental cost effectiveness ratio (ICER)^46^. It should be noted that the cost effectiveness were calculated for the treatment of rheumatoid arthritis patients. This is therefore not relevant to its effects in reducing MACE. A previous systematic review suggested that TNF inhibitors were cost effective when applying a threshold of 50,000 Euro per Quality adjusted life years (QALY) as a treatment of joint inflammation^47^. Further, cost-effectiveness of bDMARDs have been shown to be superior to csDMARDs when analysed using the number needed to treat (NNT) method^48^. With respect to the MACE, the CANTOS trial was the only large scale trial focused on secondary prevention of recurrent cardiovascular events and was estimated to increase QALY by 0.13 for a cost of $832,000, yielding an ICER of 6.4 million per QALY gained. Based on that trial Canakinumab was not considered cost-effective. Targeted use of DMARDs, particularly if they can be offered at a lower cost might lead to a cost-effective treatment but this remains to be shown^49^.

The findings of this meta-analysis should be interpreted after considering a number of limitations. The number of included trials was small limiting the statistical power. Five trials had a high risk of bias^18,33,39,40,43^ due to failure to follow intent-to-treat principles and other methodological weaknesses. The participants included were noted to have heterogeneous diagnoses which complicates the interpretation of the findings. Only one risk of bias assessment was used based on the Cochrane collaborative tool. The Grading of Recommendations, Assessment, Development and Evaluation (GRADE) criteria were not reported. Of particular note the majority of the trials did not report the medical treatments, such as anti-platelet, lipid modifying, and anti-hypertensive medications, prescribed to participants and thus it was not possible to investigate if any variation in this may have impacted on the results.

In conclusion, this meta-analysis suggested that the three DMARD sub-classes tested did not significantly affect the risk of MACE. Further trials in people at high risk of cardiovascular events are needed before firm conclusions can be drawn.

## Supplementary Information

Supplementary Information

## References

[1] Ross R (1999). Atherosclerosis—an inflammatory disease. N. Engl. J. Med..

[2] Libby P, Ridker PM, Hansson GK (2009). Inflammation in atherosclerosis: from pathophysiology to practice. J. Am. Coll. Cardiol..

[3] Singh TP (2017). Systematic review and meta-analysis of the association between C-reactive protein and major cardiovascular events in patients with peripheral artery disease. Eur. J. Vasc. Endovasc. Surg..

[4] Kaptoge S (2010). C-reactive protein concentration and risk of coronary heart disease, stroke, and mortality: an individual participant meta-analysis. Lancet.

[5] Ridker PM (2008). Rosuvastatin to prevent vascular events in men and women with elevated C-reactive protein. N. Engl. J. Med..

[6] Ridker PM (2017). Antiinflammatory therapy with canakinumab for atherosclerotic disease. N. Engl. J. Med..

[7] Aletaha D, Smolen JS (2018). Diagnosis and management of rheumatoid arthritis: a review. JAMA.

[8] Benjamin OBP, Goyal A (2019). Disease Modifying Anti-Rheumatic Drugs (DMARD).

[9] Kang EJ, Kavanaugh A (2015). Psoriatic arthritis: latest treatments and their place in therapy. Therap. Adv. Chronic Dis..

[10] Ohta H (2005). Disruption of tumor necrosis factor-α gene diminishes the development of atherosclerosis in ApoE-deficient mice. Atherosclerosis.

[11] Devlin CM (2002). Genetic alterations of IL-1 receptor antagonist in mice affect plasma cholesterol level and foam cell lesion size. Proc. Natl. Acad. Sci..

[12] Yang X (2020). Inhibition of JAK2/STAT3/SOCS3 signaling attenuates atherosclerosis in rabbit. BMC Cardiovasc. Disord..

[13] Asahina A (2016). Oral tofacitinib efficacy, safety and tolerability in Japanese patients with moderate to severe plaque psoriasis and psoriatic arthritis: a randomized, double-blind, phase 3 study. J. Dermatol..

[14] Genovese MC (2018). Safety and efficacy of upadacitinib in patients with active rheumatoid arthritis refractory to biologic disease-modifying anti-rheumatic drugs (SELECT-BEYOND): a double-blind, randomised controlled phase 3 trial. Lancet.

[15] Gordon KB (2012). A Phase III, randomized, controlled trial of the fully human IL-12/23 mAb briakinumab in moderate-to-severe psoriasis. J. Investig. Dermatol..

[16] Warren RB (2017). An intensified dosing schedule of subcutaneous methotrexate in patients with moderate to severe plaque-type psoriasis (METOP): a 52 week, multicentre, randomised, double-blind, placebo-controlled, phase 3 trial. Lancet (London, England).

[17] Li L (2015). Rates of cardiovascular disease and major adverse cardiovascular events in patients with psoriatic arthritis compared to patients without psoriatic arthritis. J. Clin. Rheumatol..

[18] Mann DL (2004). Targeted anticytokine therapy in patients with chronic heart failure: results of the randomized etanercept worldwide evaluation (RENEWAL). Circulation.

[19] Ridker PM (2019). Low-dose methotrexate for the prevention of atherosclerotic events. N. Engl. J. Med..

[20] Ryan C (2011). Association between biologic therapies for chronic plaque psoriasis and cardiovascular events: a meta-analysis of randomized controlled trials. JAMA.

[21] Westlake SL (2010). The effect of methotrexate on cardiovascular disease in patients with rheumatoid arthritis: a systematic literature review. Rheumatology (Oxford).

[22] Singh S (2019). Comparative risk of cardiovascular events with biologic and synthetic disease-modifying anti-rheumatic drugs in patients with rheumatoid arthritis: a systematic review and meta-analysis. Arthritis Care Res..

[23] Westlake SL (2011). Tumour necrosis factor antagonists and the risk of cardiovascular disease in patients with rheumatoid arthritis: a systematic literature review. Rheumatology (Oxford).

[24] Champs B (2019). Short-term risk of major adverse cardiovascular events or congestive heart failure in patients with psoriatic arthritis or psoriasis initiating a biological therapy: a meta-analysis of randomised controlled trials. RMD Open.

[25] Barnabe C, Martin B-J, Ghali WA (2011). Systematic review and meta-analysis: Anti-tumor necrosis factor α therapy and cardiovascular events in rheumatoid arthritis. Arthritis Care Res..

[26] Polachek A (2017). Risk of cardiovascular morbidity in patients with psoriatic arthritis: a meta-analysis of observational studies. Arthritis Care Res..

[27] Singh JA (2016). Biologic or tofacitinib monotherapy for rheumatoid arthritis in people with traditional disease-modifying anti-rheumatic drug (DMARD) failure: a cochrane systematic review and network meta-analysis (NMA). Cochrane Database Syst. Rev..

[28] Singh JA (2017). Biologics or tofacitinib for people with rheumatoid arthritis naive to methotrexate: a systematic review and network meta-analysis. Cochrane Database Syst. Rev..

[29] Rempenault C (2018). Metabolic and cardiovascular benefits of hydroxychloroquine in patients with rheumatoid arthritis: a systematic review and meta-analysis. Ann. Rheum. Dis..

[30] Kang Y (2019). Efficacy and safety of multiple dosages of fostamatinib in adult patients with rheumatoid arthritis: a systematic review and meta-analysis. Front. Pharmacol..

[31] Castagné B (2019). Cardiovascular safety of tocilizumab: a systematic review and network meta-analysis. PLoS ONE.

[32] Fleischmann, R., et al., *Upadacitinib versus Placebo or Adalimumab in Patients with Rheumatoid Arthritis and an Inadequate Response to Methotrexate: Results of a Phase 3, Double-Blind, Randomized Controlled Trial.* Arthritis & Rheumatology (Hoboken, N.J.), 2019. 10.1002/art.41032 31287230

[33] Burmester GR (2018). Safety and efficacy of upadacitinib in patients with rheumatoid arthritis and inadequate response to conventional synthetic disease-modifying anti-rheumatic drugs (SELECT-NEXT): a randomised, double-blind, placebo-controlled phase 3 trial. Lancet (London, England).

[34] Higgins JPT (2011). The cochrane collaboration’s tool for assessing risk of bias in randomised trials. BMJ.

[35] Hartung J, Knapp G (2001). A refined method for the meta-analysis of controlled clinical trials with binary outcome. Stat. Med..

[36] Higgins JP, Thompson SG (2002). Quantifying heterogeneity in a meta-analysis. Stat. Med..

[37] Sterne JA, Gavaghan D, Egger M (2000). Publication and related bias in meta-analysis: power of statistical tests and prevalence in the literature. J. Clin. Epidemiol..

[38] Chung ES (2003). Randomized, double-blind, placebo-controlled, pilot trial of infliximab, a chimeric monoclonal antibody to tumor necrosis factor-alpha, in patients with moderate-to-severe heart failure: results of the anti-TNF therapy against congestive heart failure (ATTACH) trial. Circulation.

[39] Weisman MH (2007). A placebo-controlled, randomized, double-blinded study evaluating the safety of etanercept in patients with rheumatoid arthritis and concomitant comorbid diseases. Rheumatology (Oxford).

[40] Emery P (2015). The first study of certolizumab pegol in combination with methotrexate in DMARD-naive early rheumatoid arthritis patients led to sustained clinical response and inhibition of radiographic progression at 52 weeks: the C-early randomized, double-blind, controlled phase 3 study. Ann. Rheum. Dis..

[41] Genovese MC (2016). Baricitinib in patients with refractory rheumatoid arthritis. N. Engl. J. Med..

[42] Dougados M (2017). Baricitinib in patients with inadequate response or intolerance to conventional synthetic DMARDs: results from the RA-BUILD study. Ann. Rheum. Dis..

[43] Genovese MC (2019). Effect of filgotinib vs placebo on clinical response in patients with moderate to severe rheumatoid arthritis refractory to disease-modifying antirheumatic drug therapy: the FINCH 2 randomized clinical trial. JAMA.

[44] Abbate A (2020). Interleukin-1 blockade inhibits the acute inflammatory response in patients with ST-segment-elevation myocardial infarction. J. Am. Heart Assoc..

[45] Sepriano A (2020). Safety of synthetic and biological DMARDs: a systematic literature review informing the 2019 update of the EULAR recommendations for the management of rheumatoid arthritis. Ann. Rheumatic Dis..

[46] Joensuu JT (2016). Cost-effectiveness of biologic compared with conventional synthetic disease-modifying anti-rheumatic drugs in patients with rheumatoid arthritis: a register study. Rheumatology.

[47] Joensuu JT (2015). The cost-effectiveness of biologics for the treatment of rheumatoid arthritis: a systematic review. PLoS ONE.

[48] Harigane K (2018). FRI0070 cost-effectiveness of biologic agents for rheumatoid arthritis was superior to traditional/conventional dmards when analysed with number needed to treat(NNT) method. Ann. Rheum. Dis..

[49] Sehested TSG (2019). Cost-effectiveness of Canakinumab for prevention of recurrent cardiovascular events. JAMA Cardiol..

