# Author Correction: Effect of disease modifying anti-rheumatic drugs on major cardiovascular events: a meta-analysis of randomized controlled trials

**DOI:** 10.1038/s41598-021-91855-3

**Published:** 2021-06-07

**Authors:** Shivshankar Thanigaimani, James Phie, Smriti Murali Krishna, Joseph Moxon, Jonathan Golledge

**Affiliations:** 1 The Queensland Research Centre for Peripheral Vascular Disease (QRC-PVD), College of Medicine and Dentistry, James Cook University, Townsville, QLD 4811 Australia; 2 The Australian Institute of Tropical Health and Medicine, James Cook University, Townsville, QLD Australia; 3 The Department of Vascular and Endovascular Surgery, Townsville University Hospital, Townsville, QLD Australia

Correction to: *Scientific Reports* https://doi.org/10.1038/s41598-021-86128-y, published online 23 March 2021

In the original version of this Article, the author name Smriti Murali Krishna was incorrectly given as Smriti Krishna.

Additionally, the Supplementary Information file previously published contained track changes.

The original Article and accompanying Supplementary Information file have been corrected.

