# Supplementary material for: Effect of disease modifying anti-rheumatic drugs on major cardiovascular events: a meta-analysis of randomized controlled trials
Source: Sci Rep. 2021 Mar 23;11:6627. doi: 10.1038/s41598-021-86128-y (PMC7987985; doi:10.1038/s41598-021-86128-y)
Supplement: Supplementary file 1 — Supplementary Information [file 41598_2021_86128_MOESM1_ESM.doc]

**Effect of disease modifying anti-rheumatic drugs on major cardiovascular events: A meta-analysis of randomized controlled trials (Supplementary material)**

**Authors:** Shivshankar Thanigaimani^1, 2^, James Phie^1, 2^, Smriti Murali Krishna^1, 2^, Joseph Moxon^1,2^, and Jonathan Golledge^1, 2, 3^

^1^ The Queensland Research Centre for Peripheral Vascular Disease (QRC-PVD), College of Medicine and Dentistry, James Cook University, Queensland, Australia

^2^The Australian Institute of Tropical Health and Medicine, James Cook University, Townsville, Queensland, Australia.

^3^The Department of Vascular and Endovascular Surgery, The Townsville Hospital, Townsville, Queensland, Australia.

**Correspondence:** Professor Jonathan Golledge, Director, Queensland Research Centre for Peripheral Vascular Disease, College of Medicine and Dentistry, James Cook University, Townsville, Queensland 4811, Australia. Fax: +61 7 4796 1401; Telephone: +61 7 4796 1417; Email: Jonathan.Golledge@jcu.edu.au

# Search terms

| Database | Search Strategy | Result |
| --- | --- | --- |
| PubMed, Cochrane & Scopus | ( TITLE-ABS-KEY ( dmard OR antirheumatic OR methotrexate OR leflunomide OR sulfasalazine OR hydroxychloroquine OR adalimumab OR mepolizumab OR etanercept OR zolimomab OR infliximab OR abatement OR rituximab OR daclizumab OR natalizumab OR sevirumab OR sibrotuzumab ) OR TITLE-ABS-KEY ( gefitinib OR baroclinic OR aegerita OR bengali OR blitz OR colterol OR preli OR fixabc OR maldi OR inflected OR pessimal OR anakinra ) AND TITLE-ABS-KEY ( cardiovascular OR hcv OR myocardial AND infarction OR min OR stroke OR death OR cardiovascular AND death AND death OR mace OR major AND adverse AND cardi* AND event* OR all$cause AND mortality ) AND TITLE-ABS-KEY ( randomi* AND controlled AND trial OR rct ) ) AND DOCTYPE ( ar ) | **PubMed - 656**  **Cochrane - 2973**  **Scopus – 1185**  **Total - 4814** |

Supplementary table 1: Cardiovascular risk factors of participants from the included trials

| **Author, Year** | **Groups** | **History of MI (%)** | **History of stroke (%)** | **Current smoking (%)** | **Hypertension (%)** | **Diabetes (%)** |
| --- | --- | --- | --- | --- | --- | --- |
| Chung et al, 2003 | Placebo (n=49) | 63.0 | NR | NR | 67.0 | 41.0 |
|  | Inflximab 5 mg/kg (n=50) | 50.0 | NR | NR | 48.0 | 28.0 |
|  | Infliximab 10 mg/kg (n=51) | 67.0 | NR | NR | 43.0 | 37.0 |
| Emery et al, 2015ᵇ | Placebo (n=219) | NR | NR | NR | NR | NR |
|  | Certolizumab pegol (n=660) | NR | NR | NR | NR | NR |
| Mann et al, 2004ᵃ | Placebo (n=682) | NR | NR | NR | 47.5 | 35.0 |
|  | Etanercept QW (n=375) | NR | NR | NR | 45.0 | 34.0 |
|  | Etanercept BIW (n=683) | NR | NR | NR | 51.5 | 38.5 |
|  | Etanercept TIW (n=308) | NR | NR | NR | 56.0 | 37.0 |
| Weisman et al, 2007 | Placebo (n=269) | 73.6 | NR | NR | 56.1 | 45.0 |
|  | Etanercept (n=266) | 80.1 | NR | NR | 63.5 | 45.9 |
| Burmester et al, 2018 | Placebo (n=221) | NR | NR | NR | NR | NR |
|  | Upadacitinib 15 mg(n=221) | NR | NR | NR | NR | NR |
|  | Upadacitinib 30 mg(n=219) | NR | NR | NR | NR | NR |
| Genovese et al, 2018 | Placebo (n=169) | NR | NR | NR | NR | NR |
|  | Upadacitinib 15 mg(n=165) | NR | NR | NR | NR | NR |
|  | Upadacitinib 30 mg(n=165) | NR | NR | NR | NR | NR |
| Genovese et al., 2016 | Placebo (n=176) | NR | NR | NR | NR | NR |
|  | Baricitinib 2mg (n=174) | NR | NR | NR | NR | NR |
|  | Baricitinib 4mg (n=177) | NR | NR | NR | NR | NR |
| Genovese et al 2019 | Placebo (n=148) | NR | NR | NR | NR | NR |
|  | Filgotinib (n=153) | NR | NR | NR | NR | NR |
|  | Filgotinib (n=148) | NR | NR | NR | NR | NR |
| Dougados et al., 2017 | Placebo (n=228) | NR | NR | NR | NR | NR |
|  | Baricitinib 2mg (n=229) | NR | NR | NR | NR | NR |
|  | Baricitinib 4mg (n=227) | NR | NR | NR | NR | NR |
| Gordon et al, 2012 | Placebo (n=484) | 1.2 | NR | 30.6 | 28.7 | 8.3 |
|  | Briakinumab (n=981) | 1.9 | NR | 31.0 | 30.8 | 10.0 |
| Ridker et l., 2017 | Placebo (N=3344) | 100 | NR | 22.9 | 79.1 | 39.9 |
|  | Canakinumab 50 mg (n=2170) | 100 | NR | 24.5 | 80.7 | 39.4 |
|  | Canakinumab 150 mg (n=2284) | 100 | NR | 23.4 | 79.4 | 41.8 |
|  | Canakinumab 300 mg (n=2263) | 100 | NR | 23.7 | 79.5 | 39.2 |
| Abbate et al., 2020 | Placebo (n=35) | NR | NR | NR | 66.0 | 43.0 |
|  | Anakinra OD (n=33) | NR | NR | NR | 39.0 | 18.0 |
|  | Anakinra TD (n=31) | NR | NR | NR | 64.0 | 29.0 |
| ᵃData taken as average of two trials presented together | | | | | | |

# Supplementary table 2: Study characteristics

| **Citation** | **Sample size (Screened/ randomised/ completed)** | **Number of patients lost to follow up** | **Number of study centres** | **Inclusion criteria** | **Exclusion criteria** | **Frequency visits/Follow up** | **Primary outcome** | **Other outcomes** |
| --- | --- | --- | --- | --- | --- | --- | --- | --- |
| Chung et al, 2003 | 150/150/141 | NR | 32 US study centres | At least 18 years old with stable New York Heart Association (NYHA) class III or IV heart failure. | Major cardiovascular problems, infection, tuberculosis, previously treated with TNF inhibitors | Infliximab 5 mg/kg, infliximab 10 mg/kg (to maximum of 1 g), or placebo immediately after randomization and again at 2 and 6 weeks after randomization. Total of 3 visits. follow-up evaluations at 1, 2, 6, 10, 14, 20, and 28 weeks | Change in clinical status at 14 weeks | Changes in inflammatory markers and combined risk of death or hospitalization for worsening heart failure at 28 weeks |
| Genovese et al., 2019 | 668/449/446 | Placebo(1), Filgotinib 100mg (1), Filgotinib 200mg (1) | 104 sites (Multiple countries) | Diagnosed with RA (2010 ACR/EULAR criteria for RA), and are ACR functional class I-III), ≥ 6 swollen joints, Ongoing treatment with a stable prescription of 1 or 2 csDMARDs | Previous treatment with any janus kinase (JAK) inhibitor | Evaluations at week 4, 12 and 24 | Proportion of Participants who Achieve an American College of Rheumatology (ACR) 20% Improvement (ACR20) Response at Week 12 | Proportion of Participants who Achieve Disease Activity Score based on 28 joints (DAS28), and change from Baseline in the Health Assessment Questionnaire - Disability Index (HAQ-DI) Score |
| Emery et al, 2015 | 880/879/643 | Placebo (6), Intervention (14) | 181 sites (Multiple countries) | Rheumatoid Arthritis (RA) less than 1 year, DMARD-naïve. | Inflammatory arthritis, History of infected joint prosthesis, Tuberculosis | Every 2 weeks | Percentage of Subjects in Sustained Remission at Week 52 | Percentage of Subjects in Sustained Low Disease Activity (LDA) at Week 52 |
| Mann et al, 2004 | 2048/2048/1090 | <1% in all groups | 301 (Multiple countries) | age 18 to 85 years; NYHA class II to IV; ischemic or nonischemic etiology; left ventricular ejection fraction <0.30 | Severe infection within 1 month, surgically correctable causes of heart failure, other serious illness, acute myocardial infarction or hospitalization (3 months), and recent (3 months) or planned surgery/coronary revascularization. | The median time from randomization to the last visit was 5.7 months in RECOVER and 12.9 months in RENAISSANCE. | Composite of death (of all causes) or hospitalization for or with CHF | All-cause mortality and the total number of hospitalizations and emergency room visits for or with worsening CHF, as well as, at 24 weeks, change in NYHA class, patient global assessment, and quality of life |
| Weisman et al, 2007 | 564/564/535 | NR | Forty-eight sites in the United States | At least 18 years of age, met the American College of Rheumatology criteria for RA [3], and had at least one qualifying comorbidity: diabetes mellitus or chronic pulmonary disease in the preceding year | Recent myocardial infarction, angina pectoris, uncontrolled hypertension or severe pulmonary disease requiring continual oxygen therapy, those under TNF antagonist therapy | Evaluations were performed at baseline, weeks 8 and 16, and 30 days post–therapy | Incidence of medically important infections | None |
| Burmester et al, 2018 | 1083/661/618 | Placebo (1), intervention (2) | 150 sites in 35 countries | 18 years or older with RA for >3 months and had an inadequate response to at least one csDMARDs | Inadequate response to bDMARDs, any previous exposure to a JAK inhibitor, or history of inflammatory joint diseases other than rheumatoid arthritis, serum aspartate transaminase or serum alanine transaminase concentrations more than two times the upper limit of normal | Efficacy, patient-reported outcomes, laboratory assessments, adverse event assessments were measured at weeks 1, 2, 4, 8, and 12 | Achieved a 20% improvement in the ACR criteria and DAS28(CRP) of 3.2 or less at week 12 | Achieved 50% or 70% improvement in the ACR criteria and DAS28(CRP) less than 2∙6 at week 12 |
| Genovese et al, 2018 | 778/499/451 | Placebo (3), Intervention (0) | 153 sites in 26 countries. | 18 years or older, rheumatoid arthritis and inadequate response or intolerance to bDMARDs, and were receiving concomitant background conventional synthetic DMARDS (csDMARDs) | Prior exposure to any Janus kinase (JAK) inhibitor. History of any arthritis with onset prior to age 17 years or current diagnosis of inflammatory joint disease other than RA. | Efficacy, patient-reported outcomes, laboratory assessments, adverse event assessments were measured at weeks 1, 2, 4, 8, and 12 | Proportions of patients achieving a 20% improvement in ACR20 at week 12 and achieving a 28-joint disease activity score using DAS28 [CRP] of 3·2 or less at week 12. | Change From Baseline in in Disease Activity Score 28 (CRP) at Week 12. Change From Baseline in Health Assessment Questionnaire Disability Index (HAQ-DI) at Week 12 |
| Gordon et al, 2012 | 1865/1465/766 | Placebo (5), Intervention (17) | 116 sites in USA and Canada | Aged 18 or above with moderate to severe plaque psoriasis | Pregnant or breast-feeding women, prior anti-IL-12 exposure, active skin conditions interfering with psoriasis evaluation, poorly controlled medical condition, hepatic, renal, or hematologic diseases, malignancy history, other than successfully treated basal cell carcinoma or non-metastatic SCC, or cervical carcinoma, and infections or risk for severe infections | Intervention every four weeks | Primary analyses conducted by non-responder imputation compared proportions achieving PGA ‘‘clear/minimal’’ (weeks 12 and 52) | Secondary analyses compared PASI 75, PASI 90, and PASI 100 response rates over time, and proportions achieving a DLQI of ‘‘0.’’ |
| Ridker et l., 2017 | 17482/10061/10049 | Placebo (7), intervention 50mg (4), 150mg (3), 300mg (4) | 1133 (Multiple countries) | Myocardial infarction and had a blood level of high-sensitivity C-reactive protein of 2 mg or more per litre despite the use of aggressive secondary prevention strategies | History of chronic or recurrent infection, previous cancer other than basal-cell skin carcinoma, a suspected or known immunocompromised state, a history or high risk of tuberculosis or disease related to the human immunodeficiency virus, or ongoing use of other systemic anti-inflammatory treatments | All doses of canakinumab and placebo were administered subcutaneously once every 3 months; for the 300-mg dose, the regimen was 300 mg every 2 weeks for the first two doses, then once every 3 months | The primary efficacy end point was the first occurrence of nonfatal myocardial infarction, any nonfatal stroke, or cardiovascular death in a time-to-event analysis | Hospitalization for unstable angina that led to urgent revascularization. And, Death from any cause and the composite of nonfatal myocardial infarction, any nonfatal stroke, or death from any cause |
| Abbate et al., 2020 | 311/99/77 | 0 | 2 sites in USA | Acute STEMI or planned coronary angiogram and under 21 years | Pregnancy, congestive heart failure, left ventricular dysfunction, severe valvular heart disease or active infections or malignancies | NA | Acute Phase Response (CRP Levels) at 14 days | Left Ventricular End-systolic Volume [ Time Frame: 12 months ] |
| Genovese et al., 2016 | 959/527/446 | Baricitinib 4mg (n=1) | 103 sites multiple countries | Had moderately to severely active rheumatoid arthritis (≥6 tender joints of 68 joints examined, ≥6 swollen joints of 66 joints examined, and a serum C-reactive protein level ≥3 mg per litre) | Recent, clinically significant infection or treatment for tuberculosis started within 4 weeks were excluded | Patients completing the study could enter a long-term extension study or were seen for follow-up approximately 28 days after the end of the treatment period | Percentage of Participants Achieving American College of Rheumatology 20% (ACR20) Response | Health Assessment Questionnaire–Disability Index (HAQ-DI) score, the 28-joint Disease Activity Score based on C-reactive protein level (DAS28-CRP) |
| Dougados et al., 2017 | 1241/684/611 | placebo (n=1) | 147 sites in multiple countries | Active RA (≥6/68 tender and ≥6/66 swollen joints; serum high sensitivity C-reactive protein (CRP) ≥3.6 mg/L (upper limit of normal 3.0 mg/L)) | Currently on steroids, NSAIDS or previous use of biologic DMARD | Patients completing the 24-week study either entered a long-term extension study or were followed for ∼28 days | Percentage of Participants Achieving American College of Rheumatology 20% (ACR20) Response | Disease Activity Score (DAS28) and Simplified Disease Activity Index (SDAI) score ≤3 |

## Supplementary table 3: Leave one out analysis for major adverse cardiovascular events in trials testing JAK inhibitors

| **Study excluded** | **I^2^ (%)** | **Risk ratio (95% confidence interval)** |
| --- | --- | --- |
| Genovese 2019 | 0 | 0.79 (0.14,4.28) |
| Dougados 2017 | 0 | 1.11 (0.50,2.44) |
| Genovese 2016 | 0 | 0.60 (0.12,3.01) |
| Genovese 2018 | 0 | 0.59 (0.12,2.95) |
| Burmester 2018 | 0 | 0.67 (0.12,3.86) |

## Supplementary table 4: Leave one out analysis for major adverse cardiovascular events in trials testing interleukin inhibitors

| **Study excluded** | **I^2^ (%)** | **Risk ratio (95% confidence interval)** |
| --- | --- | --- |
| Gordon 2012 | 0 | 0.87 (0.30,2.59) |
| Abbate 2020 | 0 | 1.08 (0.00,3.05*10^2^) |
| Ridker 2017 | 0 | 1.10 (0.00,4.22*10^4^) |

## Supplementary table 5: Leave one out analysis for myocardial infarction in trials testing JAK inhibitors

| **Study excluded** | **I^2^ (%)** | **Risk ratio (95% confidence interval)** |
| --- | --- | --- |
| Genovese 2019 | 0 | 0.50 (0.00,3.41*10^3^) |
| Dougados 2017 | 0 | 0.99 (0.88,1.12) |
| Genovese 2016 | 0 | 0.50 (0.00,2.98*10^3^) |

## Supplementary table 6: Leave one out analysis for myocardial infarction in trials testing interleukin inhibitors

| **Study excluded** | **I^2^ (%)** | **Risk ratio (95% confidence interval)** |
| --- | --- | --- |
| Abbate 2020 | 0 | 0.90 (0.08,10.68) |
| Ridker 2017 | 0 | 1.50 (0.04,63.17) |
| Gordon 2012 | 0 | 0.86 (0.71,1.04) |

## Supplementary table 7: Leave one out analysis for stroke in trials testing JAK inhibitors

| **Study excluded** | **I^2^ (%)** | **Risk ratio (95% confidence interval)** |
| --- | --- | --- |
| Genovese 2019 | 0 | 0.71 (0.23,2.17) |
| Dougados 2017 | 0 | 0.71 (0.23,2.19) |
| Genovese 2016 | 0 | 0.50 (0.14,1.83) |
| Genovese 2018 | 0 | 0.50 (0.14,1.80) |
| Burmester 2018 | 0 | 0.50 (0.14,1.83) |

## Supplementary table 8: Leave one out analysis for stroke in trials testing IL inhibitors

| **Study excluded** | **I^2^ (%)** | **Risk ratio (95% confidence interval)** |
| --- | --- | --- |
| Abbate 2020 | 0 | 0.93 (0.86,1.01) |
| Gordon 2012 | 0 | 0.76 (0.02,31.41) |
| Ridker 2017 | 0 | 0.92 (0.43,1.98) |

# Supplementary table 9: Leave one out analysis for death by any cause in trials testing TNF inhibitors

| **Study excluded** | **I^2^ (%)** | **Risk ratio (95% confidence interval)** |
| --- | --- | --- |
| Mann 2004 | 0 | 1.26 (0.03,60.40) |
| Emery 2015 | 0 | 1.09 (0.41,2.95) |
| Chung 2003 | 0 | 1.06 (0.49,2.29) |

# Supplementary table 10: Leave one out analysis for death by any cause in trials testing JAK inhibitors

| **Study excluded** | **I^2^ (%)** | **Risk ratio (95% confidence interval)** |
| --- | --- | --- |
| Dougados 2017 | 0 | 1.02 (0.87,1.18) |
| Genovese 2018 | 12 | 0.39 (0.00,3.49*10^4^) |
| Genovese 2016 | 15 | 0.40 (0.00,4.12*10^4^) |

# Supplementary table 11: Leave one out analysis for death by any cause in trials testing IL inhibitors

| **Study excluded** | **I^2^ (%)** | **Risk ratio (95% confidence interval)** |
| --- | --- | --- |
| Abbate 2020 | 0 | 0.94 (0.91,0.97) |
| Gordon 2012 | 0 | 0.52 (0.00,1.78*10^3^) |
| Ridker 2017 | 4 | 0.79 (0.00,1.59*10^2^) |

## Supplementary table 12: Leave one out analysis for serious or severe infection in trials testing TNF inhibitors

| **Study excluded** | **I^2^ (%)** | **Risk ratio (95% confidence interval)** |
| --- | --- | --- |
| Weismann 2007 | 0 | 1.10 (0.33,3.66) |
| Mann 2004 | 0 | 1.05 (0.25,4.37) |
| Emery 2015 | 0 | 1.05 (0.27,4.07) |
| Chung 2003 | 0 | 0.92 (0.74, 1.14) |

## Supplementary table 13: Leave one out analysis for serious or severe infection in trials testing JAK inhibitors

| **Study excluded** | **I^2^ (%)** | **Risk ratio (95% confidence interval)** |
| --- | --- | --- |
| Genovese 2019 | 0 | 1.16 (0.47,2.83) |
| Dougados 2017 | 0 | 1.29 (0.59,2.85) |
| Genovese 2016 | 0 | 1.20 (0.44,3.26) |
| Genovese 2018 | 0 | 0.98 (0.59,1.63) |
| Burmester 2018 | 0 | 1.05 (0.48,2.27) |

## Supplementary table 14: Leave one out analysis for serious or severe infection in trials testing IL inhibitors

| **Study excluded** | **I^2^ (%)** | **Risk ratio (95% confidence interval)** |
| --- | --- | --- |
| Gordon 2012 | 0 | 1.09 (0.93,1.29) |
| Ridker 2017 | 72 | 0.39 (0.00,6.59*10^5^) |
| Abbate 2020 | 79 | 0.43 (0.00,1.28*10^6^) |

# Supplementary Table 15: Quality assessment using Cochrane tool

| **Author** | **Clearly defined initial diagnoses of patient subset** | **Random sequence was generation** | **Mentioned the number of patients who completed the study** | **Blinding of participants/personnel** | **Blinding of assessors** | **Sample size estimate clearly mentioned** | **Incomplete outcome measure (>10% loss)** | **Primary outcome was clearly defined** | **ITT** | **Other biases** |
| --- | --- | --- | --- | --- | --- | --- | --- | --- | --- | --- |
| **TNF inhibitors** | | | | | | | | | | |
| Chung et al, 2003 | Patients with stable New York Heart Association class III or IV heart failure and left ventricular ejection fraction <35% | Eligible patients were randomly assigned in a double-blind fashion | Yes | Yes | Yes | Assuming that the proportions of placebo patients with improved, unchanged, and worsened clinical status at week 14 were similar to those observed in the 47-patient etanercept trial (45%, 42%, and 13%, respectively), a sample size of 150 patients would have been sufficient to detect rates in the combined infliximab groups of 75%, 15%, and 10%, respectively, with a power of 80%. | None | Yes | ITT | None |
| Genovese et al., 2019 | Patients with RA (2010 ACR/EULAR criteria for RA), and are ACR functional class I-III. | Patient randomization was stratified based on the number of previous bDMARDs and the analysis was pre-specified to examine the number of prior treatments | Yes | Yes | Yes | Sample size was targeted to provide 90% power at a 2-sided .05 level to detect a difference of 0.25 between Filgotinib and placebo on the change from baseline in HAQ-DI at week 12 and to provide more than 90% power to detect an increase in ACR20 response rate of 25% to 45% between placebo and Filgotinib groups | None | Yes | ITT | None |
| Emery et al, 2015 | Fulfilled the 2010 American College of Rheumatology (ACR)/European League Against Rheumatism (EULAR) classification criteria | Randomisation at Week 0 was performed centrally using an interactive voice and web response system and was stratified by disease duration of >4 months or ≤4 months | Yes | Yes | Yes | Assumed an expected percentage of patients in sustained remission (sREM) at Week 52 of 50% in the CZP+MTX group and 30% in the PBO+MTX group. A minimum of 600 CZP patients and 200 PBO patients were required (for 3:1 randomisation) | <1% | Yes | NR | None |
| Mann et al, 2004 | Yes. Patients with New York Heart Association class II to IV chronic heart failure and a left ventricular ejection fraction <.30. | In RECOVER trial, the patients were randomized on a 1:1:1 basis to placebo or etanercept 25 mg SC once weekly (QW) or twice weekly (BIW), whereas in RENAISSANCE trial, patients were randomized to placebo or etanercept 25 mg BIW or 25 mg 3 times weekly (TIW) | Yes | Yes | Yes | Using a stratified log-rank analysis, 389 events would provide 90% power for a 2-sided 0.01 level test to detect a 32.4% relative reduction in risk for this end point, when the combined BIW and TIW etanercept treatment arms were compared with placebo. To control the overall type I error rate within each study at 0.05, analyses of the clinical status composite were conducted at the 2-sided 0.04 level, and the combined analysis of all-cause mortality or CHF hospitalization was conducted at the 2-sided 0.01 level. | None | Yes | NR | A single independent Data Monitoring Committee (DMC) monitored and recommended modifications to the protocols to enhance patient safety or quality of trial conduct |
| Weisman et al, 2007 | Yes, patients who met the American College of Rheumatology criteria for RA | Randomization was stratified by diagnosis of diabetes (with or without another comorbidity) and the diabetic stratum was further stratified by treatment with oral hypoglycaemic agents or insulin (or both therapies). Patients in each stratum were randomized in a 1:1 ratio to subcutaneous injections of placebo or etanercept (25 mg) twice weekly for 16 weeks. | Yes | Yes | Yes | The planned sample size (1000 patients) was based on literature estimates of infections in the general population. If the incidence of MIIs in the placebo group was 10% and the true relative risk was 2.0 (i.e. incidence of 20% in the etanercept group), the study design provided 84% power to detect the difference between treatment groups. | None | Yes | NR | An independent data safety monitoring board (DSMB) was established before study initiation to periodically review unblinded safety data, recommend protocol modifications and propose early study termination if persuasive evidence was observed for futility or for harm or benefit attributable to etanercept |
| **JAK inhibitors** | | | | | | | | | | |
| Burmester et al, 2018 | Yes. Fulfilled the 2010 American College of Rheumatology (ACR) and European League Against Rheumatism (EULAR) classification criteria for rheumatoid arthritis | Interactive response technology with a randomisation schedule generated by the data sciences department at AbbVie | Yes | Yes | Yes | Planned a sample size of 600 patients to provide 90% power for a 21% difference in ACR20 and a 22% difference in DAS28(CRP) of 3∙2 or less, assuming placebo responses of 37% for ACR20 and 15% for DAS28(CRP) of 3∙2 or less, with a two-sided α of 0∙025, and accounting for a 10% dropout rate | None | Yes | NR | None |
| Genovese et al, 2018 | Yes. Fulfilled the 2010 American College of Rheumatology (ACR) and European League Against Rheumatism (EULAR) classification criteria for rheumatoid arthritis | Interactive response technology | Yes | Yes | Yes | Planned a sample size of 450 patients to provide a 90% power for a 20% difference in ACR20 and a 17% difference in DAS28(CRP) of 3∙2 or less, assuming placebo responses of 27% for ACR20 and 12% for DAS28(CRP) of 3∙2 or less, at a two-sided α of 0∙025 level of significance and accounting for a 10% dropout rate | None | Yes | ITT | None |
| Genovese et al., 2016 | Moderate to severely active rheumatoid arthritis (≥6 tender joints of 68 joints examined, ≥6 swollen joints of 66 joints examined, and a serum C-reactive protein level ≥3 mg per liter). | Random assignment in a 1:1:1 ratio for each group | Yes | Yes | Yes | Estimated that a sample size of approximately 175 patients per study group would provide 90% or greater power for the comparison of the ACR20 response rate between the 4-mg baricitinib group and the placebo group (with assumed rates of 45% and 25%) at week 12 | None | Yes | ITT | None |
| Dougados et al., 2017 | Active RA (≥6/68 tender and ≥6/66 swollen joints; serum highsensitivity C-reactive protein (CRP) ≥3.6 mg/L (upper limit of normal 3.0 mg/L)) | Random assignment in a 1:1:1 ratio for each group | Yes | Yes | Yes | Estimates determined that 220 patients per treatment group would provide >95% power for comparison between baricitinib 4 mg and placebo in ACR20 response rate (assumed 60% vs 35%, respectively) at week 12. | None | Yes | ITT | None |
| **Interleukin inhibitors** | | | | | | | | | | |
| Gordon et al, 2012 | Unclear. Moderate to severe psoriasis, however, no guidelines cited | Randomization was administered via an interactive voice response system, thus concealing treatment allocation from all study personnel and subjects | Yes | Yes | Yes | A sample size of 1,350 patients (randomized 2:1, briakinumab: placebo) was estimated to provide 490% power to demonstrate the superiority of briakinumab over placebo. Assuming that 630 briakinumab treated patients would be re-randomized 2:2:1 at week 12, this sample size would provide >90% power to detect an 18% treatment difference | None | Yes | ITT | Assessment of primary outcome is subject to observer error. And the percentage of participants who completed the study is very low. |
| Ridker et l., 2017 | Yes, patients with history of MI | Used a centralized computer system, with stratification according to the time since the index myocardial infarction and according to trial part | Yes | Yes | Yes | Assuming that all three active doses would result in a primary event rate that was 20% lower than the rate with placebo, we calculated that the trial would have more than 90% power to detect a significantly lower risk with at least one canakinumab dose than with placebo | <1% | Yes | ITT | It is not clear whether MI and stroke data are either fatal or non-fatal only |
| Abbate et al., 2020 | Acute STEMI defined as chest pain (or equivalent) with an onset within 12 hours and ECG evidence of ST segment elevation (>1 mm) in 2 or more anatomically contiguous leads that is new or presumably new | Randomization log with allocation concealment | Yes | Yes | Yes | Yes | None | Yes | ITT | No information on previous use of biologic DMARDs |

# Supplementary Figures


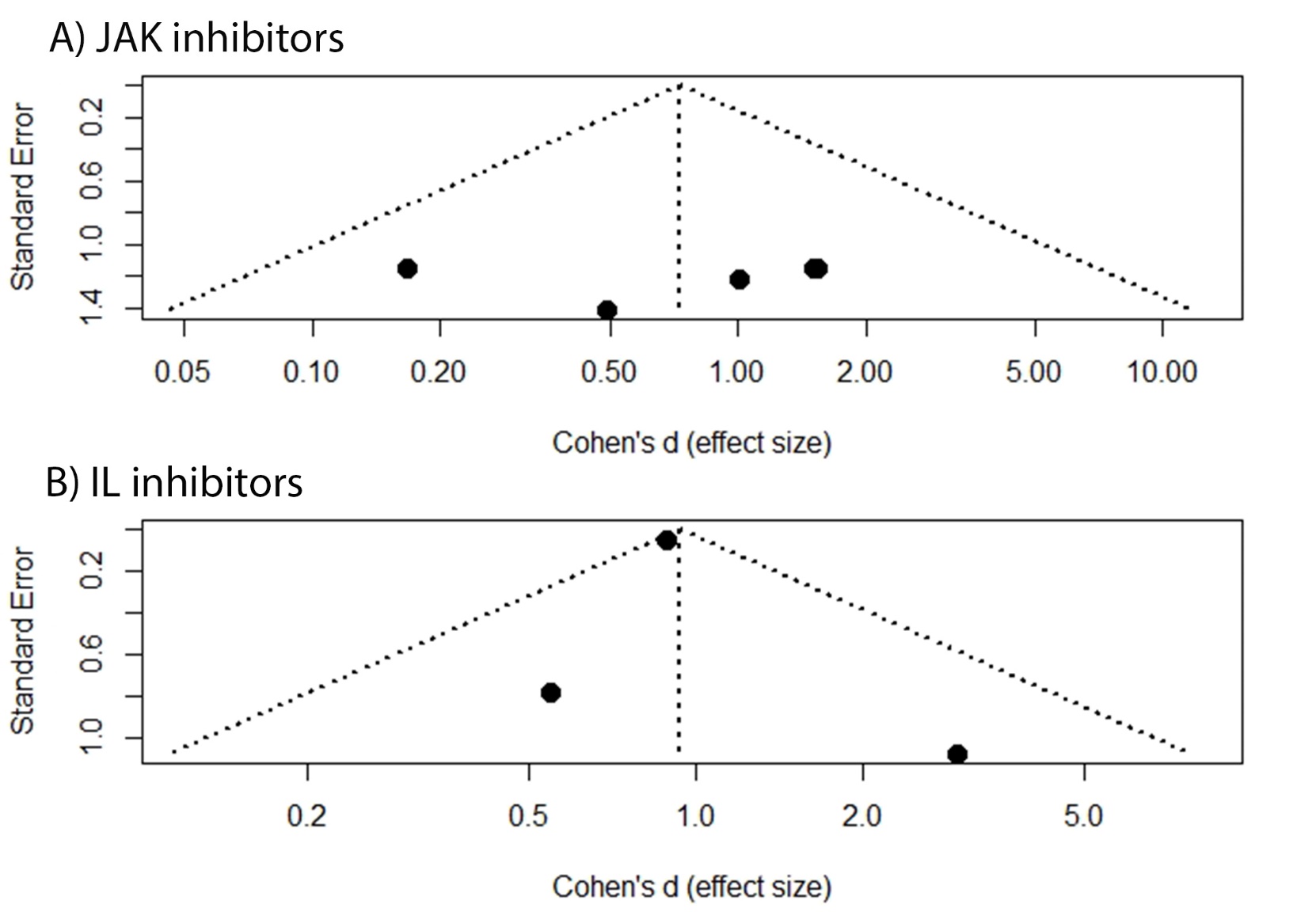


Supplementary figure 1: Funnel plot of studies examining major adverse cardiovascular events


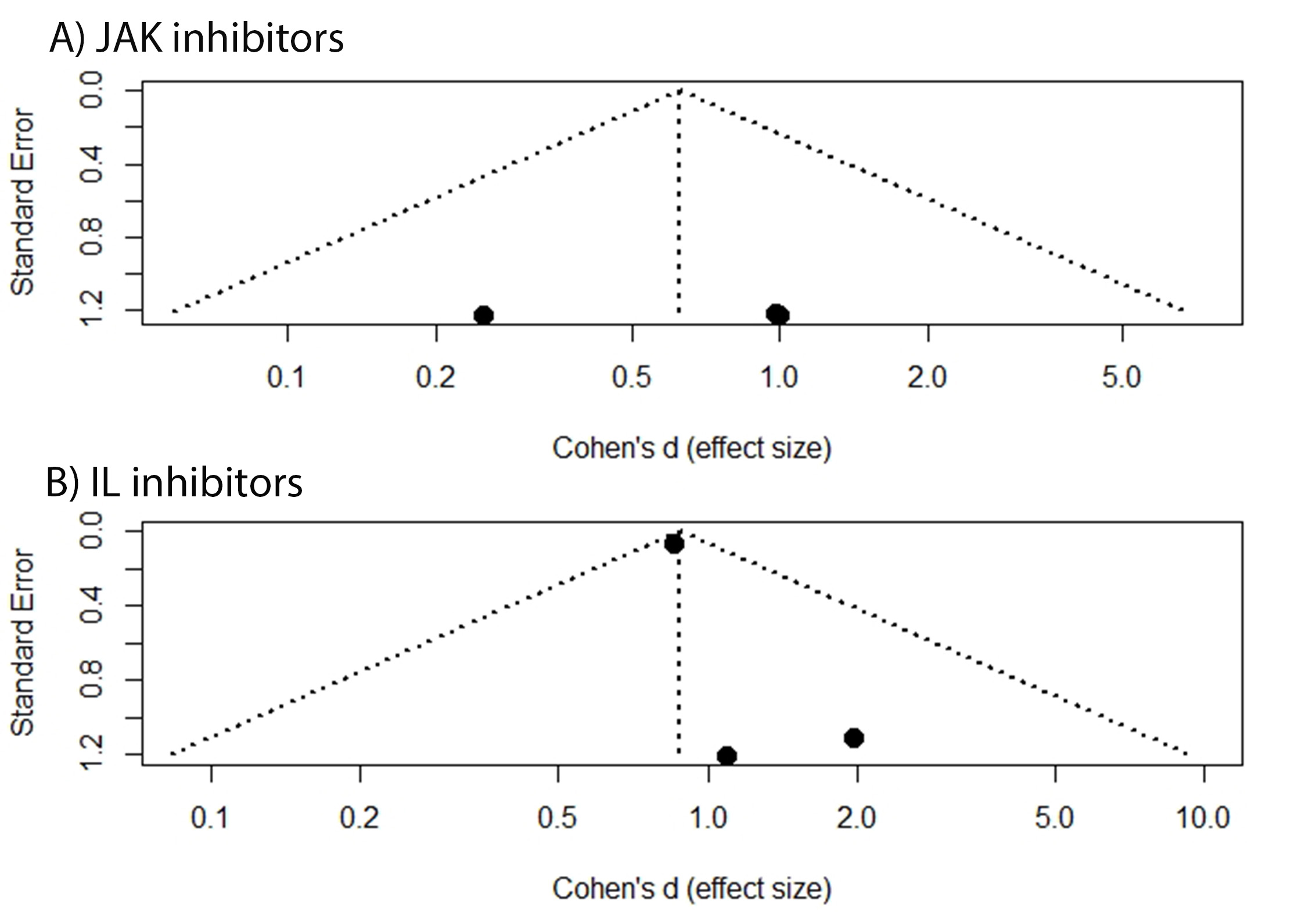


*Supplementary figure 2: Funnel plot of studies examining myocardial infarction alone*


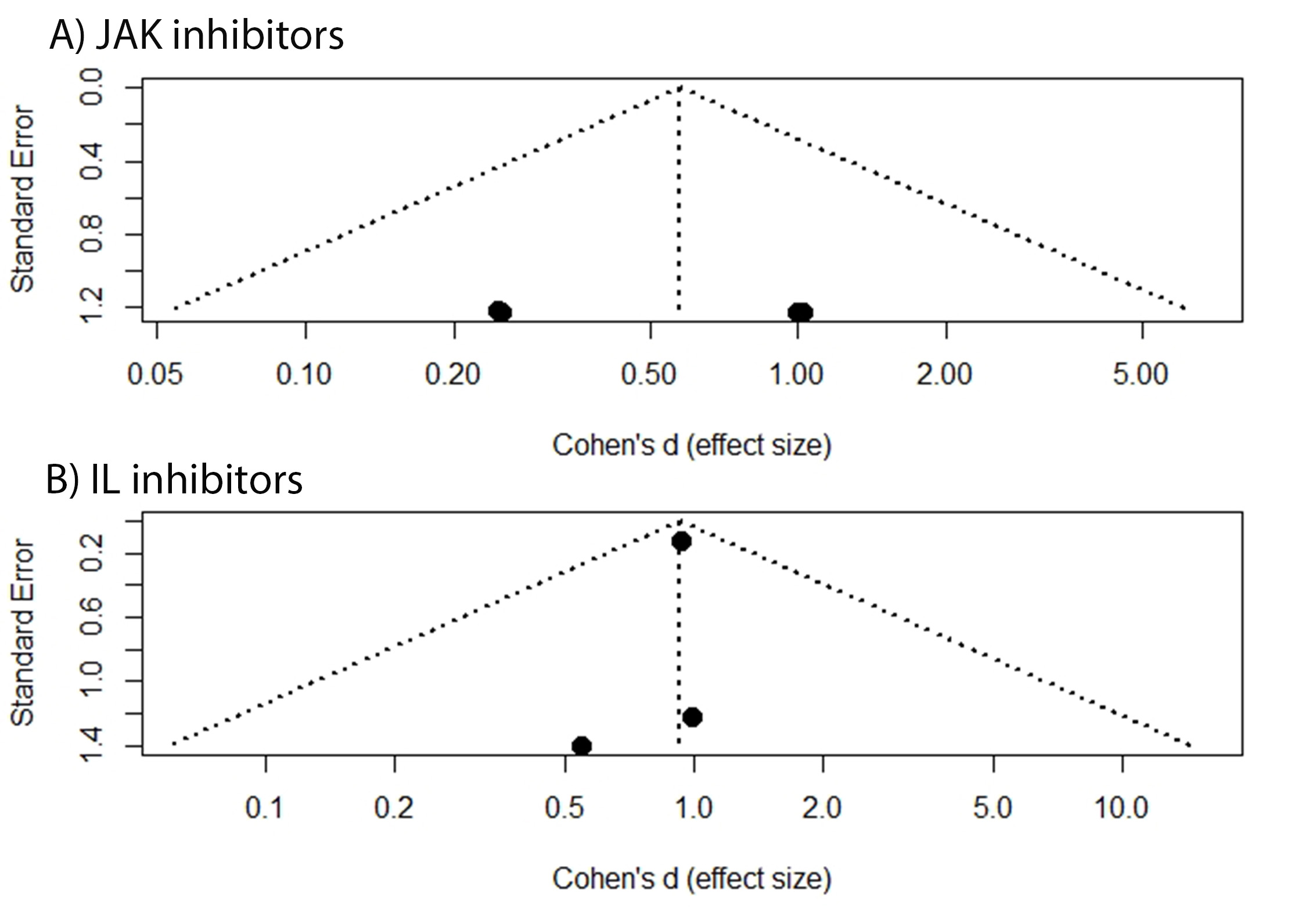


### *Supplementary figure 3: Funnel plot of studies examining stroke alone*


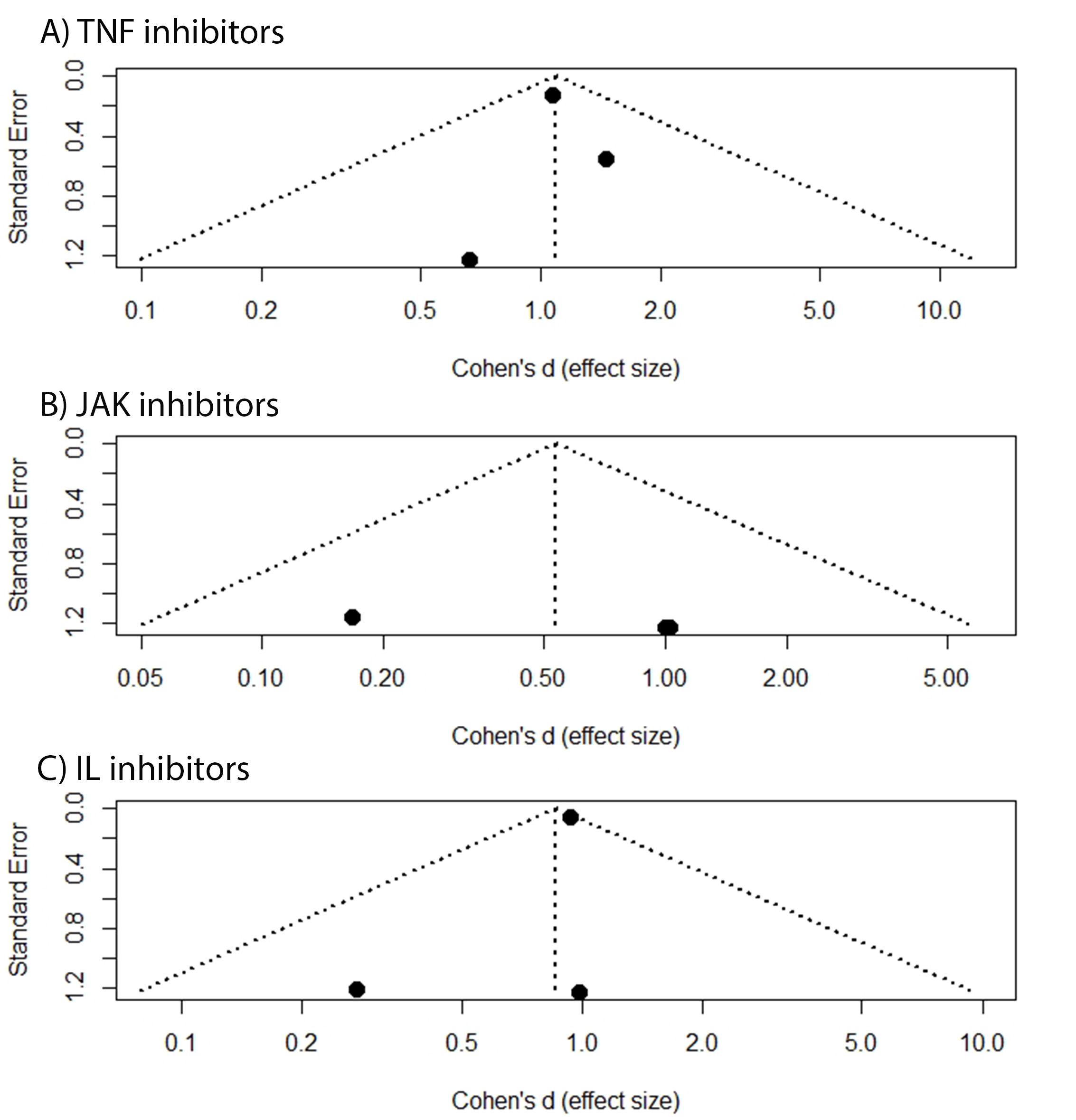


### *Supplementary figure 4: Funnel plot of studies examining death by any cause*


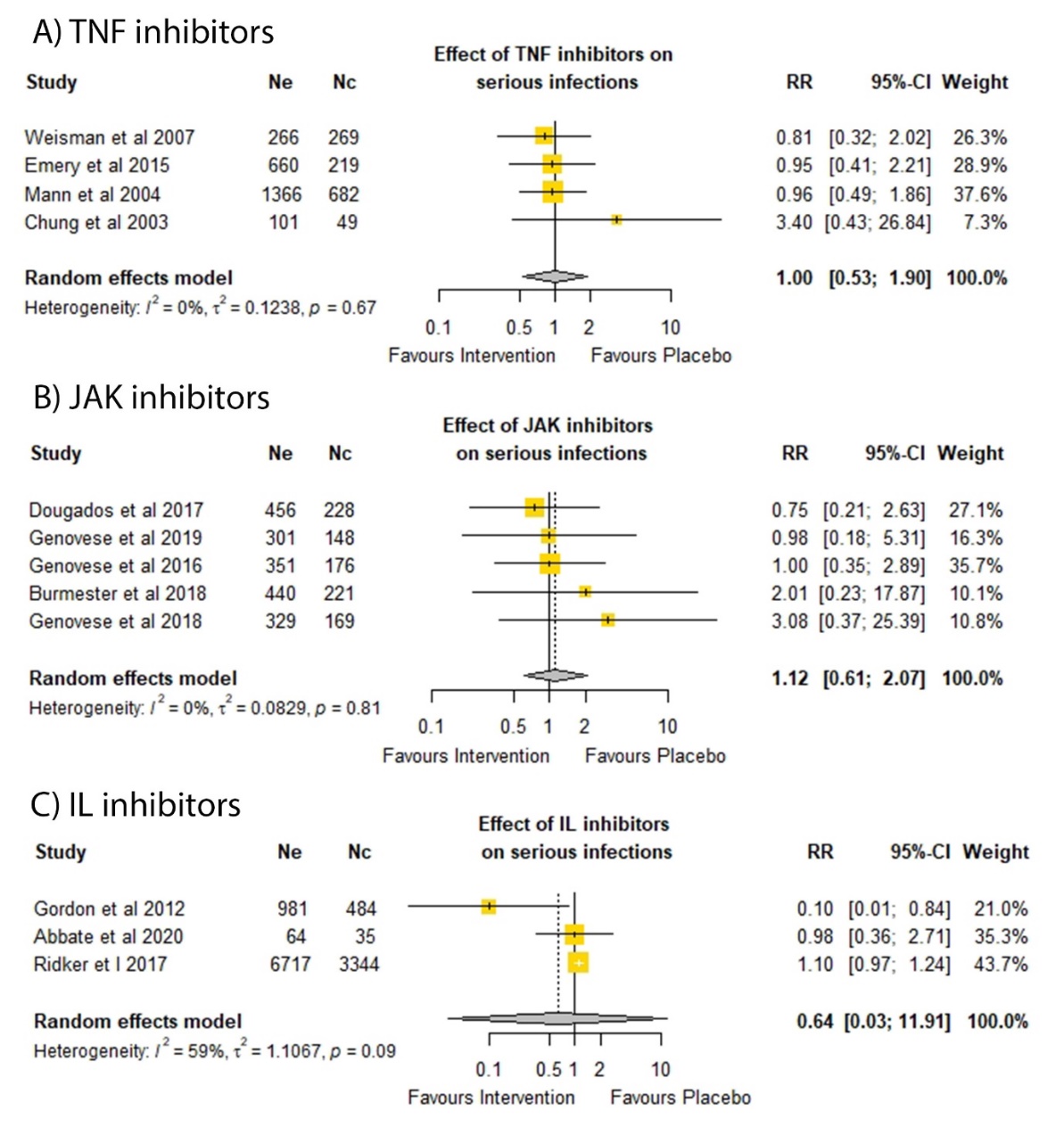


Supplementary figure 5: Effect of DMARDs on serious or fatal infections.

*
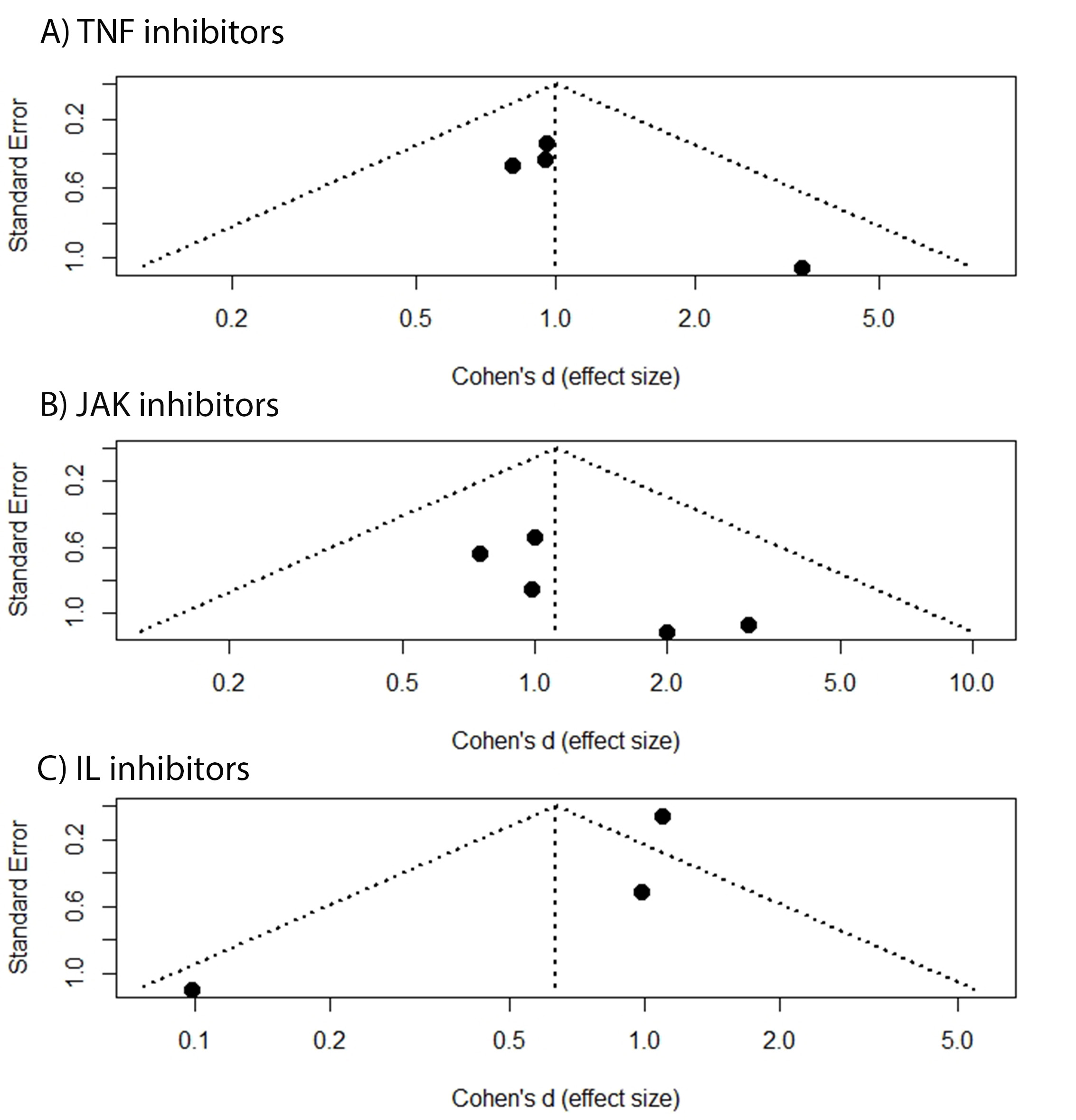
*

### *Supplementary figure 6: Funnel plot of studies examining serious or severe infections*
